# Archaeological and anthropological studies on the Harappan cemetery of Rakhigarhi, India

**DOI:** 10.1371/journal.pone.0192299

**Published:** 2018-02-21

**Authors:** Vasant S. Shinde, Yong Jun Kim, Eun Jin Woo, Nilesh Jadhav, Pranjali Waghmare, Yogesh Yadav, Avradeep Munshi, Malavika Chatterjee, Amrithavalli Panyam, Jong Ha Hong, Chang Seok Oh, Dong Hoon Shin

**Affiliations:** 1 Department of Archaeology, Deccan College Post-Graduate and Research Institute, Pune, India; 2 Lab of Bioanthropology, Paleopathology and History of Diseases, Institute of Forensic Science/ Department of Anatomy, Seoul National University College of Medicine, Seoul, South Korea; 3 Department of Oral Biology, Division in Anatomy & Developmental Biology, BK21 PLUS Project, Yonsei University College of Dentistry, Seoul, Republic of Korea; Max Planck Institute for the Science of Human History, GERMANY

## Abstract

An insufficient number of archaeological surveys has been carried out to date on Harappan Civilization cemeteries. One case in point is the necropolis at Rakhigarhi site (Haryana, India), one of the largest cities of the Harappan Civilization, where most burials within the cemetery remained uninvestigated. Over the course of the past three seasons (2013 to 2016), we therefore conducted excavations in an attempt to remedy this data shortfall. In brief, we found different kinds of graves co-existing within the Rakhigarhi cemetery in varying proportions. Primary interment was most common, followed by the use of secondary, symbolic, and unused (empty) graves. Within the first category, the atypical burials appear to have been elaborately prepared. Prone-positioned internments also attracted our attention. Since those individuals are not likely to have been social deviants, it is necessary to reconsider our pre-conceptions about such prone-position burials in archaeology, at least in the context of the Harappan Civilization. The data presented in this report, albeit insufficient to provide a complete understanding of Harappan Civilization cemeteries, nevertheless does present new and significant information on the mortuary practices and anthropological features at that time. Indeed, the range of different kinds of burials at the Rakhigarhi cemetery do appear indicative of the differences in mortuary rituals seen within Harappan societies, therefore providing a vivid glimpse of how these people respected their dead.

## Introduction

Harappan Civilization, named after the first site discovered close to the village of Harappa (Punjab, Pakistan), has been examined and appreciated since the early twentieth century because this is the earliest complex society known from ancient South Asia. The cultures of the Harappan Civilization can generally be subdivided into Early (3300~2600 BCE), Mature (2600~1900 BCE), and Late (1900~1700 BCE) periods [1]. Recent excavations in the Ghaggar Basin (or *RgVedic Saraswati*) sites, including Bhirrana, Girawad, Farmana, and Rakhigarhi, have pushed the date for the beginning of the Harappan Civilization back to 5500 BCE [2]. The significance and geographical extent of this civilization are now clearer than ever as it encompassed a vast area spanning southeastern Afghanistan and Pakistan, as well as the northwestern and western states of India [3].

According to the relevant previous literatures [3,4], this civilization was originally formed as the result of the gradual development of indigenous farming communities. Their eventual unification was the beginning of a complex urban society. Because of extensive inter-community trade networks, Harappan people shared a common cultural tradition characterized by life in well-planned and organized towns or cities. They boasted multiple hallmarks of an advanced civilization such as copper-bronze metallurgical techniques, a standard measurement system, shared ceramic idioms, a written language and so on. To date, five major urban sites (Mohenjo-daro, Harappa, Ganweriwala, Rakhigarhi, and Dholavira), each originally surrounded by a vast rural landscape and small settlements, have been identified as regional centers of Harappan Civilization [3,5].

**Fig 1:**
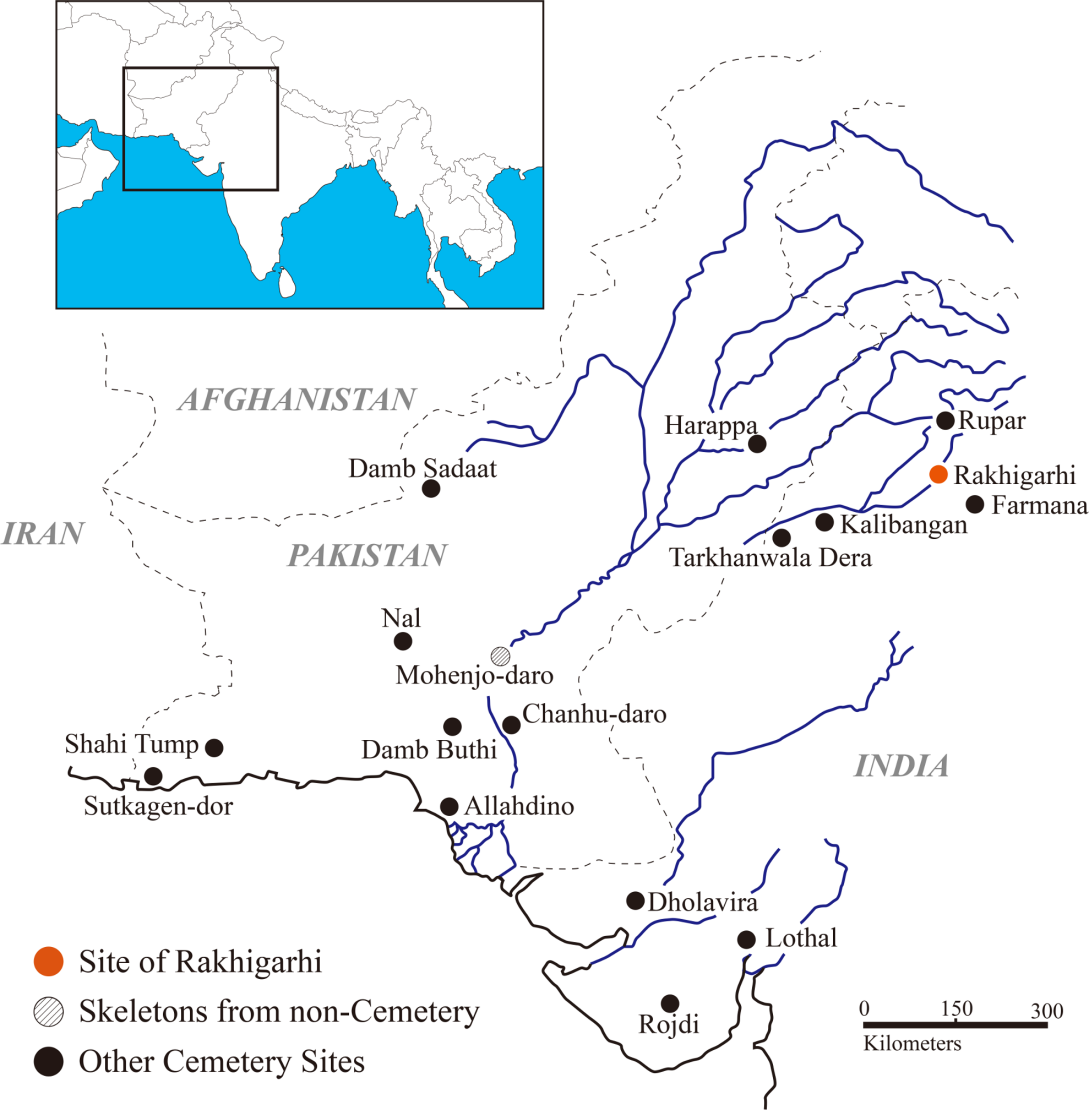
Harappan sites where skeletons were discovered (indicated by dots). Red dot: Rakhigarhi site; dashed dot: skeletons from non-cemetery area; black dots: cemetery sites other than Rakhigarhi.

Over the last 100 years, archaeologists have uncovered a number of Harappan cemetery sites (Fig 1), including Harappa [6,7], Kalibangan [8], Farmana [9,10], Rakhigarhi [11], and Sanauli [12]. However, the data from these sites are currently too incomplete to describe how the Harappan people treated their dead in the cemeteries [13–15]. Archaeological efforts on known Harappan cemeteries have also been limited because of their remote locations and the apparently random nature of sites. A further complicating factor has been the action of hydrological and wind erosion flattening the soil pits of burial mounds. As a result, the majority of the archaeological surveys completed on more than 2,000 sites so far have been focused mainly on Harappan cities and towns, while relatively few cemetery sites have been addressed [3].

Despite these difficulties, some pioneering interdisciplinary studies successfully reconstructed the people’s lives of Harappan Civilization, with the data from mortuaries and skeletal remains of particular interest to archaeologists and anthropologists [16–18]. Briefly speaking, the biological relationships between Harappan societies and their neighboring civilizations were revealed in previous works [19,20]. Isotopic analysis elucidated individual migration life histories linking city populations to hinterland groups [21]. Dietary [22,23] and pathological [24–27] features have also been the subject of interdisciplinary researches. Paleopathological studies were done on the teeth and mandibles of the Harappan people [26,27]. Schug et al. [24] compared the cranial traumas seen in historical populations at Harappa burials to evaluate whether the society was characterized by a peaceful heterarchy [24]. Schug [28] speculated that the presence of non-normative burials as well as traumatic injuries and leprosy in skeletal remains might have related to differences among the dead at Harappa. Other recent publications have dealt with the significance of mortuary behavior in the sociocultural dynamics of Harappan Civilization [21,26–30].

Excavations over three consecutive years (between 1997 and 2000) carried out by the Archaeological Survey of India (ASI) uncovered the evidences of a well-established road, drainage system, large rainwater storage facility, and additional city infrastructure in Rakhigarhi site [31–33]. The ASI thus established that Rakhigarhi, once surrounded by fertile cropland and numerous settlements, was the provincial capital of the eastern region of the Harappan Civilization [34]. Although this preliminary ASI fieldwork paved the way for future investigations, the majority of graves within the cemetery still remained untouched except for 11 burials in a trench within the cemetery area (RGR 07) [11]. This subsequent lack of archaeological and anthropological focus on the cemetery area has been unfortunate, especially since the Rakhigarhi site was one of the greatest political and economic centers of the Harappan Civilization.

Our investigations carried out between 2013 and 2016 at Rakhigarhi cemetery might therefore prove meaningful. By the excavation of a salvage trench in 2013–14, we were able to reveal the general features of this cemetery. We continued our excavation in the following year (2014–15), and extended its range further in the year after (2015–16). The results of this three-year study have enabled us to conjecture how the people of Harappan Civilization were buried and how their graves were managed within the necropolis. The numerous novel aspects about Harappan mortuary customs are also discussed in this paper.

## Materials and methods

### Archaeology

**Fig 2:**
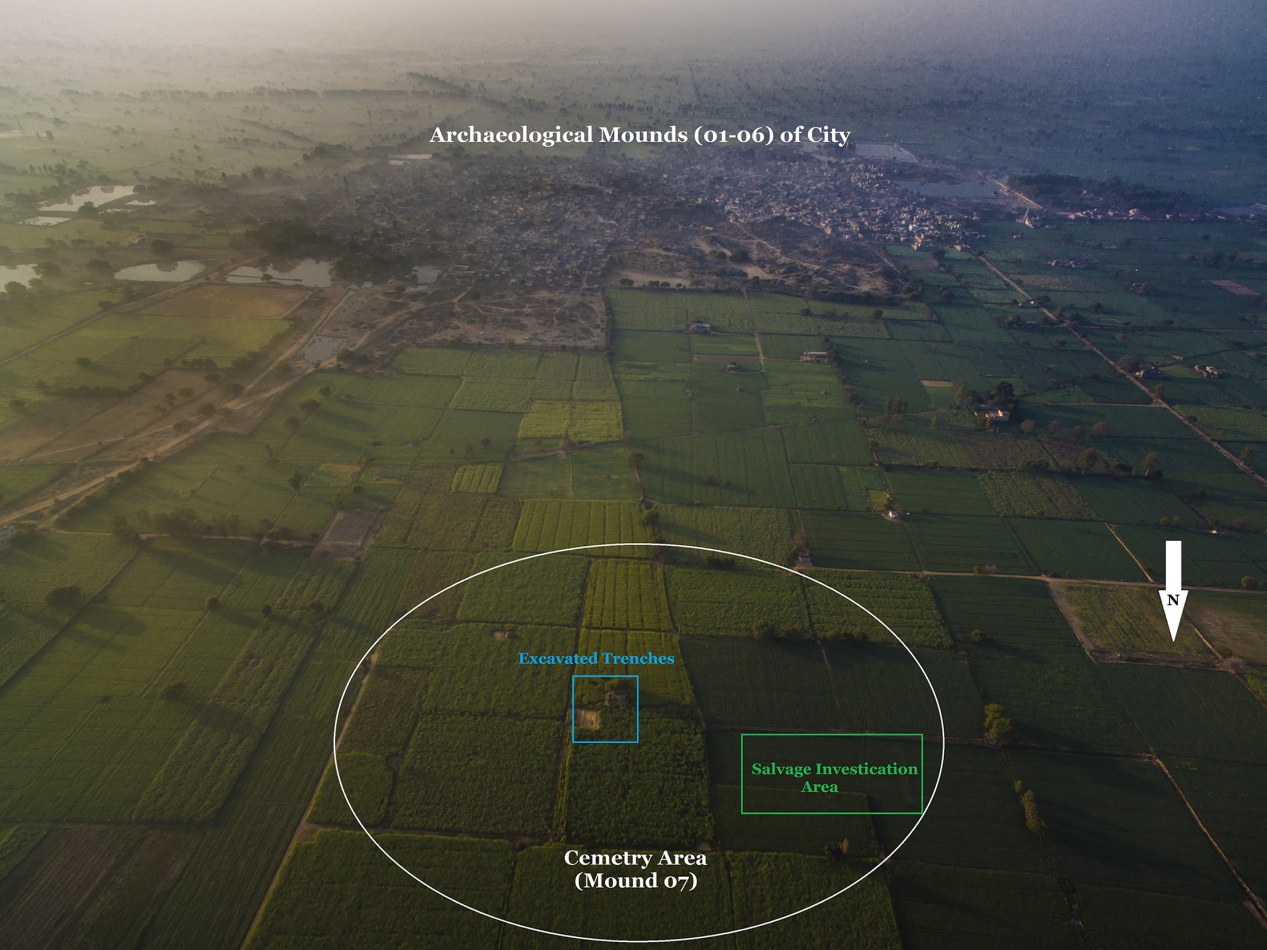
Aerial view of Rakhigarhi. Mounds 01–06: Archaeological mounds of Harappan city; Mound 07: cemetery area. The blue and green rectangles indicate the currently excavated trenches (2014–16) and salvage investigation area (2013–14), respectively.

**Fig 3:**
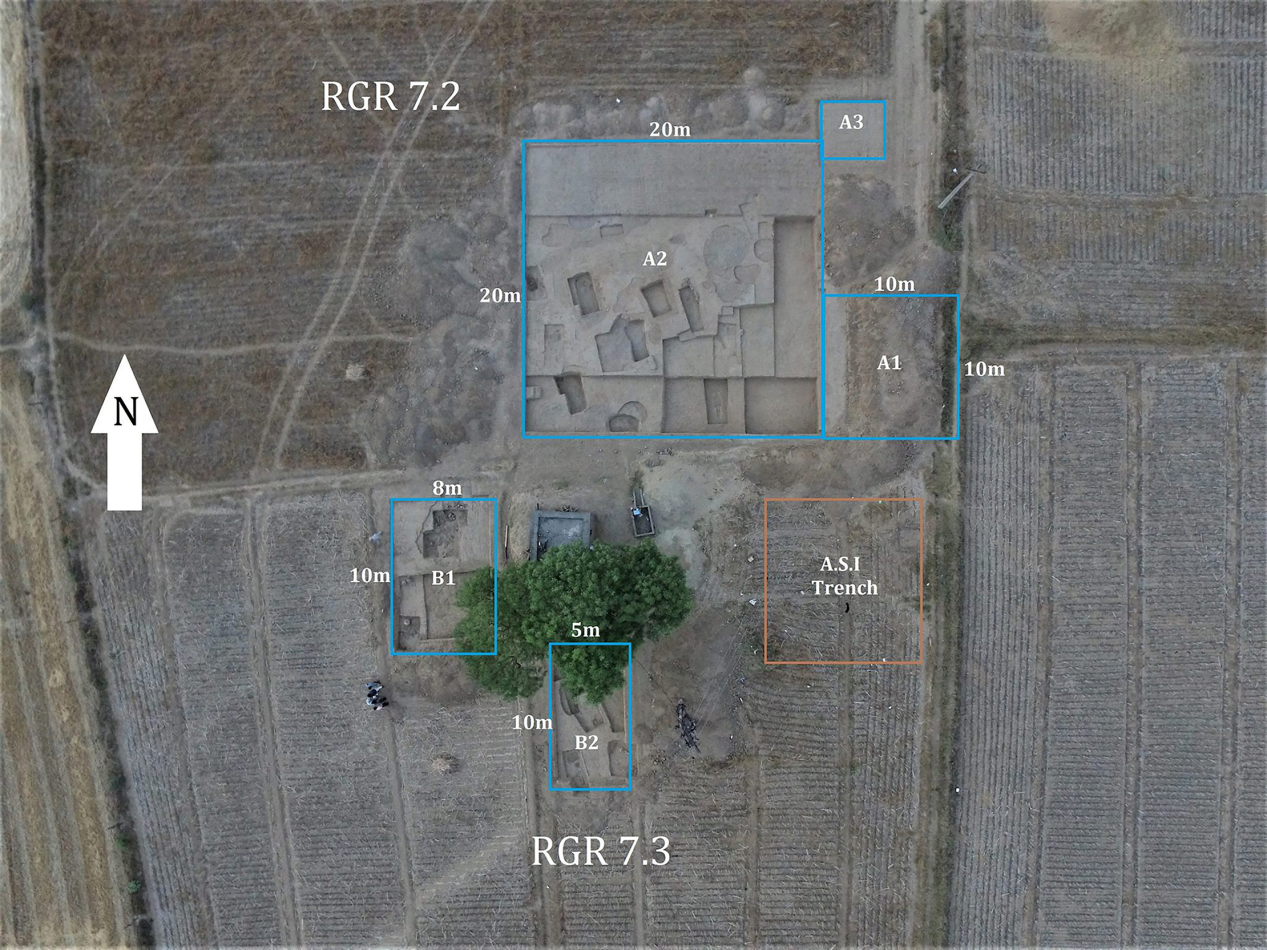
Distinct localities of Rakhigarhi excavation site.

Rakhigarhi is an ancient megacity site located about 150 kilometers from Delhi in India’s Haryana state. Its necropolis area (N29°17′52.9″/E76°06′51″) is situated in what is now an agricultural field (ASI designation: RGR 07) (Fig 2). We differentiated the area into three distinct localities: RGR 7.1 (for salvage-trench), RGR 7.2 (northern section), and RGR 7.3 (southern section) (Fig 3). In each locality, we numbered trenches and burial pits in their order of excavation (S1 Fig). We put one salvage-trench (S1 Fig) in RGR 7.1, and three (A1 to A3) and two (B1 to B2) trenches in RGR 7.2 and 7.3, respectively.

The investigation was conducted under the permission of the Archaeological Survey of India (approval number: F/15/1/2010 EE). During the excavation of the necropolis area, very large numbers of potsherds and animal bones, as suggestive of the complex mortuary activities in the cemetery area, were found around burial pits. Outside the burial pits within the trenches, common Harappan objects like hopscotch, sling balls and shell objects were collected though in small quantities. It is quite likely that these are indications of the rituals practiced by the Harappans as part of their burial ceremonies. Photos and videos were taken during and after the excavation of each burial pit. Two drones (Phantom3 Standard; Professional, DJI, Shenzhen, China) were deployed for acquisition of the aerial views of the site.

**Fig 4:**
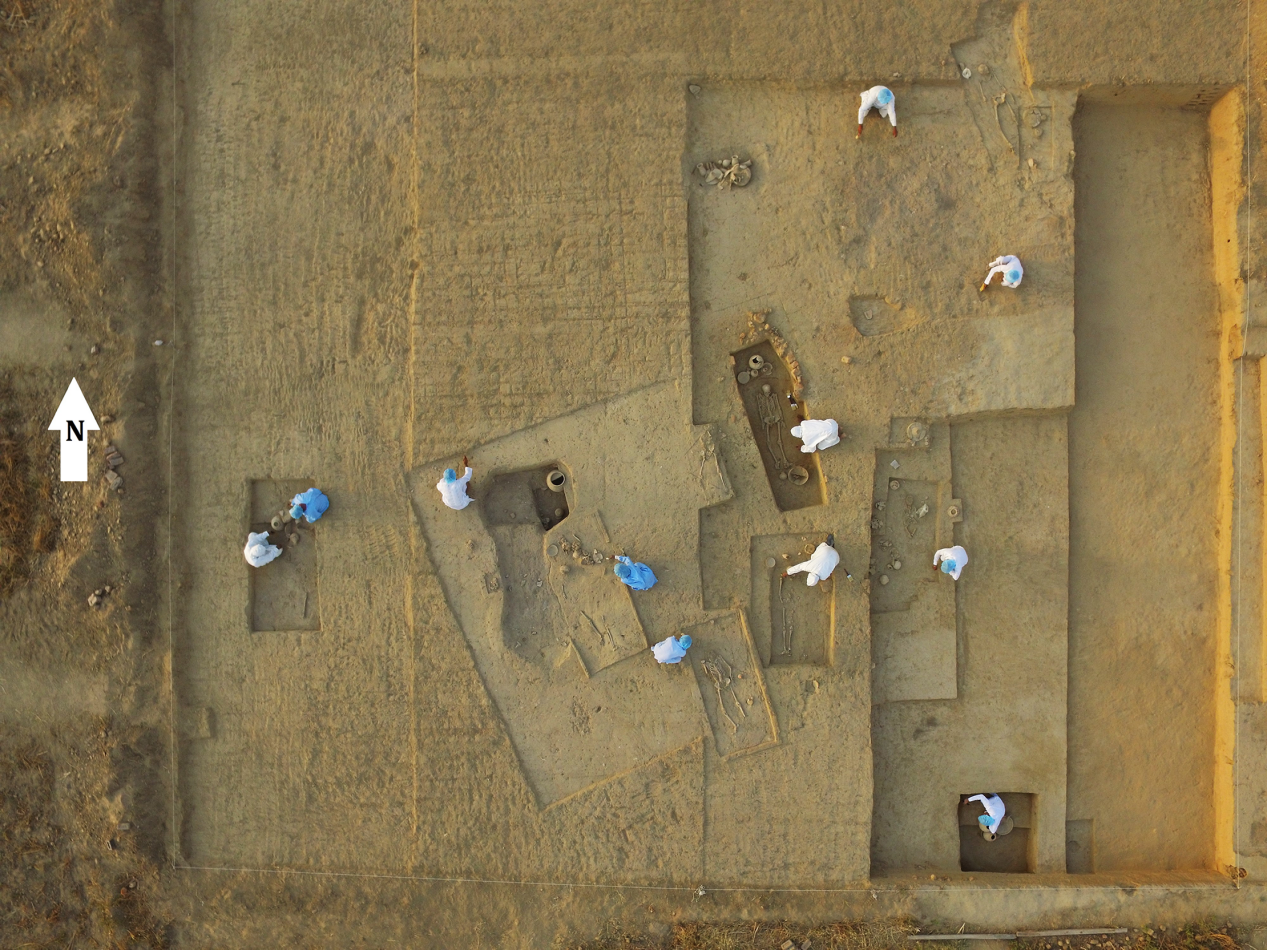
Fieldwork. Note the protective clothing worn to minimize contamination of samples.

After unearthing of skeletons at the excavation site, we recorded the relevant archaeological information. During the fieldwork, we wore protective gloves, masks, gowns and caps in order to reduce sample contamination to the minimum (Fig 4; S2 Fig). We also took steps to prevent damage to skeletons, especially by limiting access to them. For future bio-anthropological experiments, the genetic profiles of every researcher’s hair sample were obtained to compare with those of ancient specimens. The human and cultural remains retrieved from each burial pit finally were transported to the Laboratory at the Department of Archaeology, Deccan College Post Graduate and Research Institute (Deemed University), Pune, India.

The specimens discussed in this paper are housed in the collections of the Department of Archaeology, Deccan College, Post-Graduate and Research Institute, Pune, India. For the skeletons, access is available to bona fide researchers on request. The review of Institutional Review Board (IRB) for this study was exempted by Seoul National University Hospital (exemption number: 2013–004; 2017–001).

### Choosing the burial classification system

Classification is the basis of archaeological analysis. Mortuary excavation, for example, yields a wide variety of evidence reflective of practices that have to be classified systematically in terms of shared attributes and differences [35]. Concerning the archaeological aspects of disposal of the dead, early studies on ancient South Asia employed ambiguous terminology based on the assumption of the duality of burial, either inhumation or cremation [36]. We had to reject this protocol for classification of burials discovered at Harappan cemeteries, as inhumation and cremation were not mutually exclusive in practice.

Singh [37] suggested the following system of burial-type classification for South Asia: (1) burial of complete body: inhumation in pits or urns; (2) burial of selected bones after cremation: post-cremation burial, and (3) burial of selected bones after excarnation: post-excarnation burial. Although an improvement on the former protocol, this classification system cannot be considered the standard for field archaeology either, as it neglects some burial cases (e.g. a cenotaph or empty grave for commemoration of the deceased) due to the technical limitations at the time. Next, Sprague [38,39] classified body disposal into simple (primary inhumation irrespective of aquatic, superterranean or subterranean disposals) and compound (involving a reduction process before final disposal) cases. Although these definitions are consistent with primary and secondary burials, respectively, they are nonetheless too ambiguous for classification of every grave in the archaeological context of South Asia.

Nowadays, Harappan archaeologists prefer a classification system accounting for primary, secondary, and symbolic burials, each sub-categorized by the difference in the means of disposal of the body (full, fractional or absent body) [9,10,40]. This classification yields a comprehensive set of mutually exclusive categories. Primary burial indicates any method whereby the full body is interred (e.g., underground pit, built-grave, ship-burial, hanging burial, etc.) as the final stage of burial. Secondary burial represents burial of a fractional part or parts of the body that were collected after artificial or natural decomposition. Symbolic burial covers the practices whereby the grave is built at a location other than the burial place of the dead body. This classification system is beneficial to field archaeologists, as it is also applicable to other historical mortuary-archaeological contexts of the Indian subcontinent (e.g. Iron Age megalithic burial) [41]. In consideration of the aspects and advantages above-noted, we adopted this system for classification of mortuary customs evidenced in our study of Rakhigarhi cemetery.

**Fig 5:**
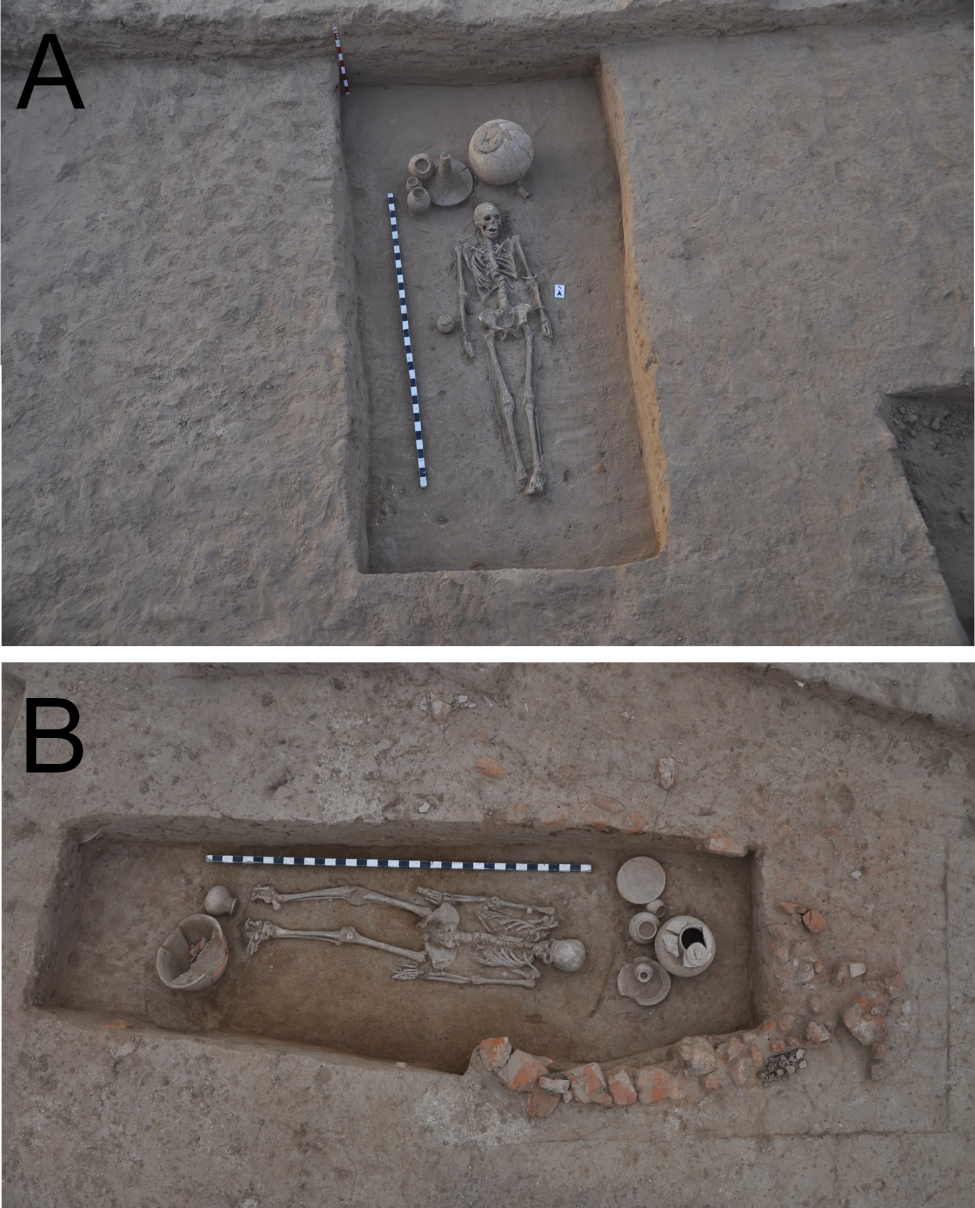
(A) Primary typical interment (A2/BR 36) at Rakhigarhi cemetery. (B) Primary atypical burial (A2/BR 22) with brick-lined grave architecture.

Specific individuals, communities and societies have their own normative methods of burial. What was or were the Harappan Civilization’s normative form or forms of body disposal remains unclear to us. And indeed, we have to allow for the possibility that diverse groups within the broader Harappan society had distinctive mortuary customs [3,5,18,42]. Such uncertainty as to what practices were normative for the Harappan Civilization make our classification fundamentally etic. We thus sub-categorized the Rakhigarhi cemetery’s primary burials into typical and atypical cases. Typical cases, entailing burial of supine-positioned bodies inside of a plain pit (Fig 5A), were found in much greater numbers than were atypical, exceptional cases such as brick-lined graves (Fig 5B), multiple bodies or prone-positioned burial. The Harappan people’s common practice was, at least as far as Rakhigarhi cemetery indicates, the burial of the body without any process of reduction.

As for secondary burial, the term is somewhat problematic due to its ambiguous usage among archaeologists. Darvill [43] defined it as involving a grave dug into a pre-existing barrow or burial at any time after its initial construction. Knüsel [44] defines secondary burial as the relocation of the body of a primary burial to another site. South Asian archaeologists, meanwhile, have their own definition of secondary burial: the final disposal of the body parts long after death, regardless of inhumation or cremation, decomposition, or earlier relocation [9,10,40]. In our study, we adopted this South Asian definition of secondary burial.

**Fig 6:**
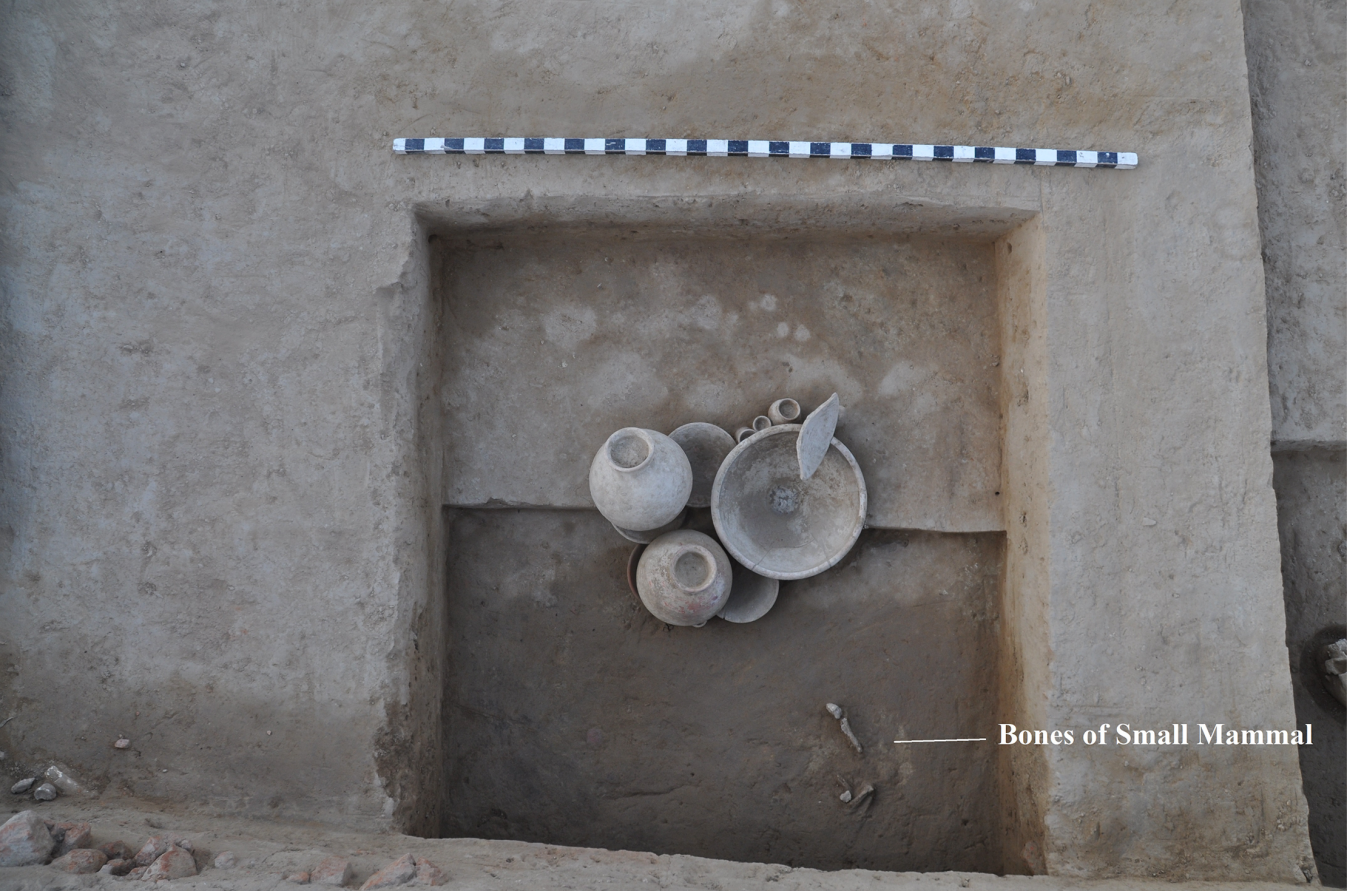
Symbolic grave (BR16).

We also categorized as symbolic burials cases in which the body was not placed inside the burial at the time that the grave was first constructed. An example is a cenotaph, a type of monument that functions as a symbolic burial to commemorate an individual whose body was missing (e.g. who died far from home) [45]. We performed careful examinations to rule out the possibility that the body had disappeared due to taphonomic agents or processes. We classified cases as symbolic when human bones were not discovered whereas sacrificed-animal bones or other grave goods were (Fig 6). Also, we classified cases as unused pit chamber when the grave had been elaborately built but absolutely no bones or artifacts were found.

### Anthropology

Anthropological studies were conducted on skeletal remains obtained from respective burial pits in order to shed light on some of the bio-anthropological characteristics of the Rakhigarhi population. During the analyses at Deccan College, archaeologists and anthropologists exchanged opinions with each other for more comprehensive understanding of the data. While the archaeologists analyzed the characteristics of each burial and grave artifacts, the anthropologists assigned the information obtained from the individual skeletons to the burial inventory archaeologists summarized.

Sex and age estimations were performed on the skeletal remains using methods described in *Standards for Data Collection* [46]. The sex estimation of the individuals was carried out by macroscopic assessments of the pelvis and skull. The primary indicators included the greater sciatic notch and pre-auricular sulcus of the pelvis. When the pelvic elements were not dispositive, the glabella, supraorbital margin, nuchal crest, and mastoid process of the skull and mandibular mental eminence were examined [46,47]. Age at death was estimated with reference to degenerative changes of the auricular and pubic symphyseal surfaces of the pelvis [48], the degrees of palatal and ectocranial suture closure [49,50], and dental wear [51]. All of the adult individuals were categorized into three age groups: young adult (18~35 years), middle-aged adult (36~50 years), and old adult (over 50 years). For immature individuals, approximate age was determined based on dental development and epiphyseal closure [52].

### Statistics

The individual’s sex, age and burial type as well as the number of votive pots were tabulated and subjected to statistical analysis using R version 3.4.0 (R Foundation for Statistical Computing, Vienna, Austria). To determine statistical difference between two independent groups, we first tested all data for normality (Shapiro-Wilk test). Next, to compare variances, we performed an F test for normally distributed groups, using Welch's t-test when the variances were not equal to each other and, in cases where they were equal, the pooled estimate of the variance. The Wilcox rank sum test for non-parametric statistical hypothesis was applied to non-normally distributed data. A P value of <0.05 was considered statistically significant (confidence interval: 95%). We drew boxplots to visualize the results by group. We also utilized Strip Charts for drawing of individual variables in a single plot. In order to detect burial outliers by the number of votive pots, we defined the inner fence as follows: Q3 + 1.5 x interquartile range (IQR). When a burial’s pot number was outside the inner fence, we regarded it as an extreme case (i.e., too divergent from the others).

## Results and discussion

### Information on excavation site

We performed a series of archaeological and anthropological analyses on the Rakhigarhi cemetery area for three consecutive seasons (2013–2016): the first season (2013–2014) for RGR 7.1, and the next two seasons (2014–2016) for full-scale excavations of RGR 7.2 and 7.3. Radiocarbon dates (determined by Accelerator Mass Spectrometry) for charcoal samples from different depths at the Rakhigarhi site were previously reported by the Inter University Accelerator Center (Delhi, India). The carbon dating of the samples at the depths of 9.1 and 20.6 meters yielded calibrated dates of 2273±38 years BCE and 2616 ±73 years BCE, respectively [53].

**Fig 7:**
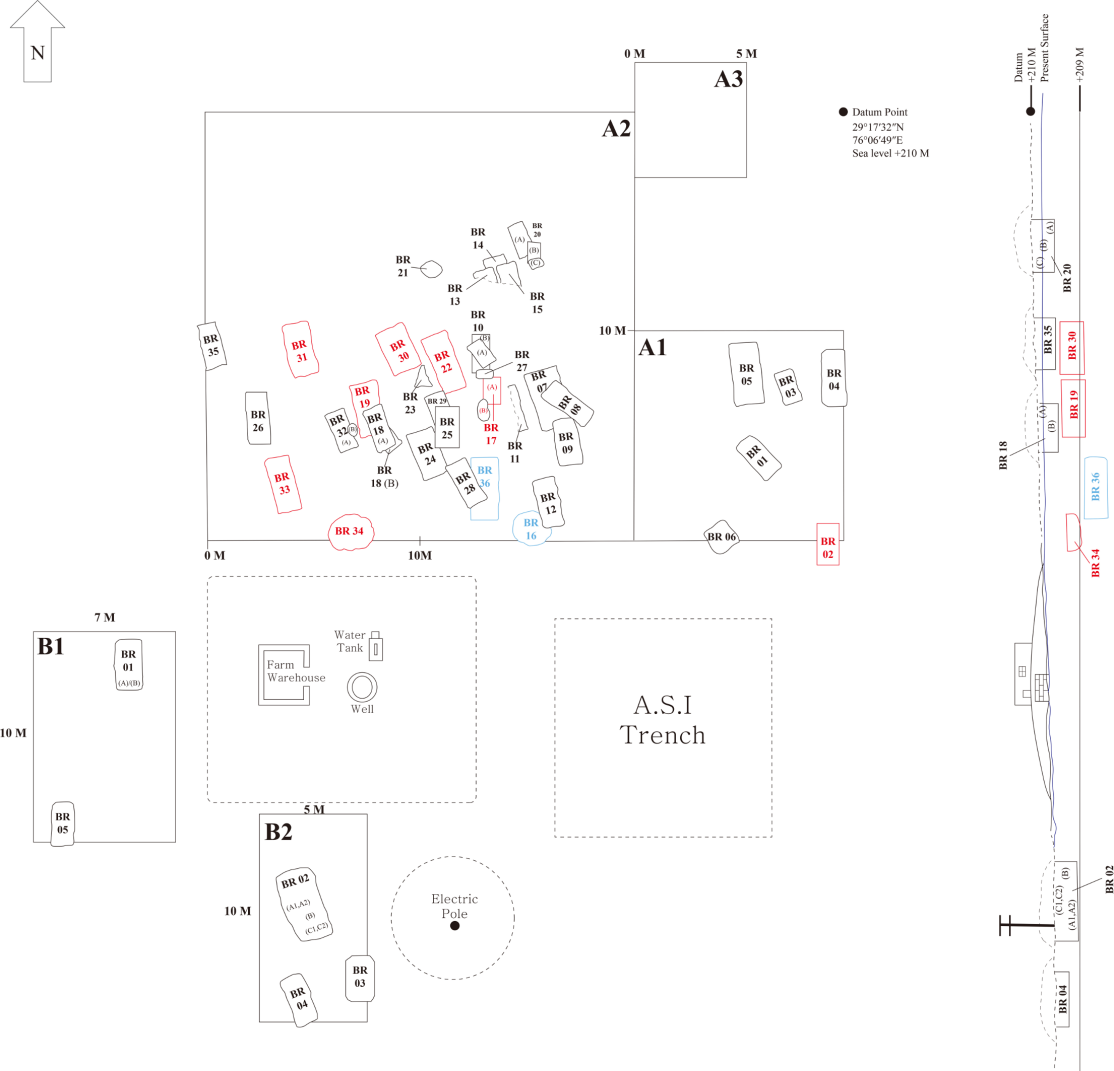
Map of excavation site at Rakhigarhi necropolis.

By surface survey and interview with village seniors (April, 2014), we were able to obtain stratigraphic information on the RGR 7.1 site. We learned, for example, that the local people had already leveled much of the mounds (about 1 meter) for farming purposes. We estimated, by surface survey of the area and its remaining portions of mounds, that the present extent of the cemetery was approximately 1 ha. In the following season (2014–15), we resumed systematic excavation of a trench (A1–10 × 10 m) in RGR 7.2, finding 6 burials (A1/BR 01–06) therein. In 2015–16, we extended the excavation area, designating a large trench (A2; 20 × 20 m) next to A1, and discovering a total of 36 burials. A small trench A3 (5 × 5 m) was assigned to check the stratigraphy of the locality. By this means, we were able to determine that the cemetery inclined from north to south at the time that the Harappan people were actively constructing graves there. The burials in the trenches within the northern locus generally remained closer to the soil surface. In the B1 to B2 trenches assigned to the southern locality (RGR 7.3), we found 11 burials: 3 in the RGR7.3 B1 trench and 8 in the B2 trench. The general information is summarized on the conceptual map of the excavation site (Fig 7).

Most of the burial pits were rectangular in shape, with vertically cut sides and flat bottoms. We also found some oval or square pits specially used for non-primary burials. Although each primary burial pit had a slightly different orientation, all were generally arranged on the north-south axis with the head to the north. The pottery and any other artifacts from all of the excavated graves belonged to the Mature Harappan period. Because the shapes of the graves and typologies of the burial goods did not much differ from each other, detailed burial chronology proved difficult.

Even so, from the information obtained by the archaeological excavations, we could classify the Rakhigarhi burials into three distinct groups (I, II and III). Group I, the earliest burials, included only two graves (BR 16 and 36) within the A2 trench. Both were found around 1.1 meters below our Datum Point. We speculate that the A2 locality might have been chosen by the Rakhigarhi people at the initial stage when the surface of the area was around -1.1 meters. There was no evidence of anthropogenic activity below this phase. The localities A1 (BR 02) and A2 (BR17A/B, 19, 22, 30, 31 and 34) became a cemetery when the surface layer was between -0.75 to -1.0 meters, at which time Rakhigarhi people were increasingly buried there. The Group II graves were found to be neatly arranged, becoming the possible ritualizing place that was constructed elaborately.

Lastly, within the A1 and A2 as well as B1 and B2 trenches and above the Group II burials, the Group III graves were found. These Group III burials showed that the cemetery had been extended to other localities (B1 and B2) beyond the earlier, focal locality (A1 and A2) where the Groups I and II burials were found. Haphazard encroachments against previously built graves (BR08 and 09 against 07, BR 13 and 15 against 14, BR 20B and C against A, BR 10A against B, BR 25 against 29, BR 18A against B) was observed to have occurred during this phase. Such carelessness, according to previous reports on other Harappan cemeteries at least, was not uncommon [7]. In summary, we conjecture that the A2 locality was chosen during the Group I phase as the first burial place, and that subsequently, during the Group II phase, the same locality (A2) became the site of greater ritualization. In the Group III phase, a well-established necropolis covering a much wider area extending beyond the A2 locality was established by the Harappan people in Rakhigarhi.

### The burials at Rakhigarhi

In the course of our three-season excavation of Rakhigarhi burials (n = 53), we deemed cases to be primary burials when the full skeleton was discovered inside a grave and there were no signs of any reduction process. These primary burials were the most commonly identified type (n = 41, 77.4%) at the Rakhigarhi necropolis.

**Fig 8:**
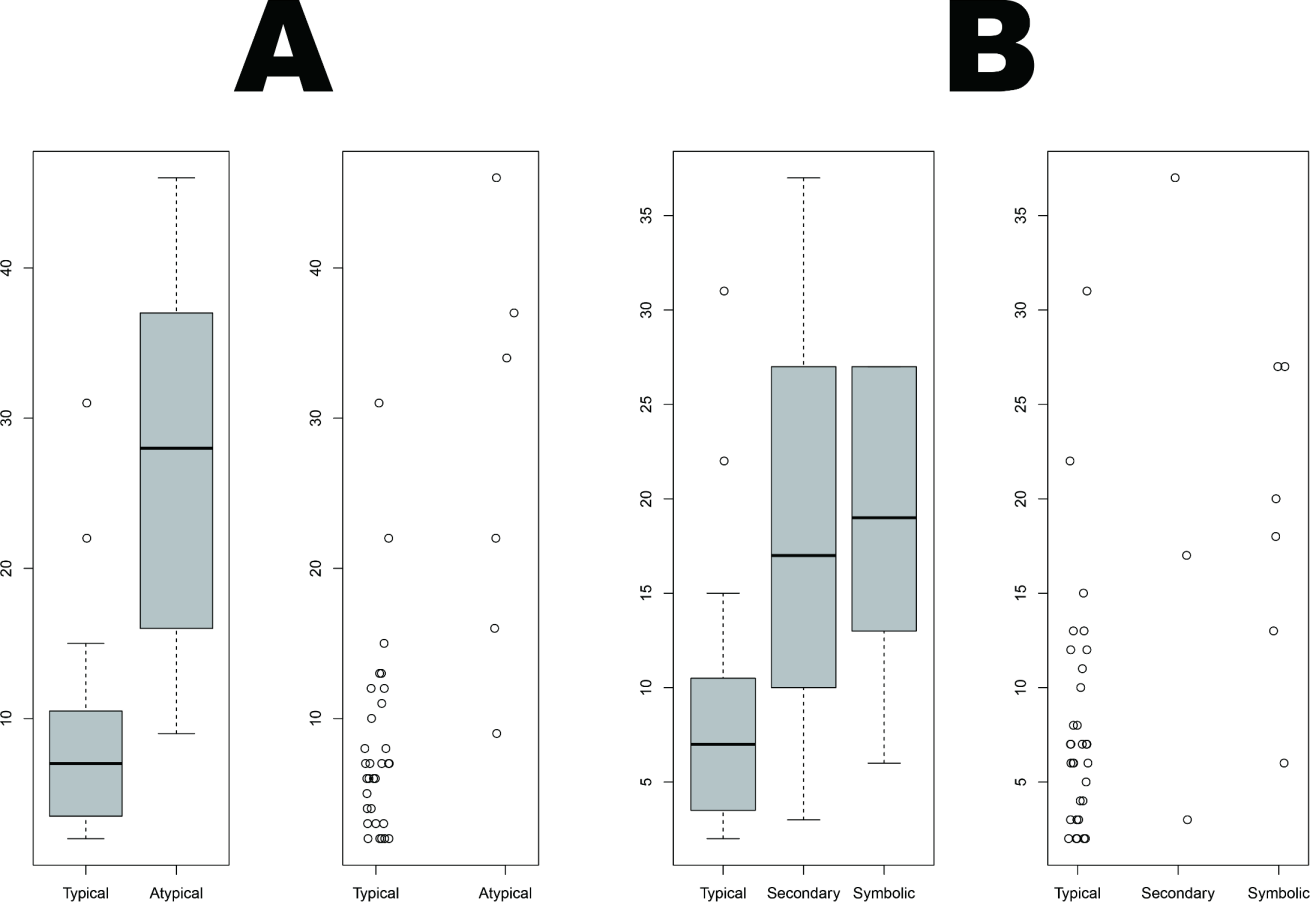
Box and scatter plots of votive pot numbers for (A) primary typical and atypical as well as (B) primary typical, secondary and symbolic graves from Rakhigarhi cemetery.

Among the primary interments, we found both typical (34/53, 64.2%) as well as atypical (7/53, 13.2%) burials. The typical burials had one characteristic in common: a singular individual buried supinely inside a simple (plain) grave. Among the primary atypical burials, on the other hand, unique patterns were exhibited, such as brick-lined grave architecture, and multiple or prone-positioned individuals inside a pit. The present study’s box plots of votive pot numbers (Fig 8A) revealed that atypical graves had significantly more votive pots than did typical graves (Wilcoxon rank sum test, W = 173.5, p = 0.0009399). Similar atypical cases were also reported from the cemetery at Harappa [7] (R-37, Mature Harappan period).

We also found, at the same cemetery, uncommon burials including secondary (5/53, 9.4%) and symbolic (6/53, 11.3%) graves (Table 1). Good examples of secondary burial at Rakhigarhi cemetery are bones inside pots buried in a circular pit (A2/BR 21) (S3 Fig). They must not have been cremated prior to burial, as they exhibited no burn marks. In most of the secondary burials, animal bones (buffalo or cattle, goat or sheep etc.) were found, either in a dish-on-stand or some other arrangement, suggesting that meat might have been offered to the dead. Additionally, there was also one unused pit chamber (1/53, 1.9%).

**Table 1:** Classification of the Harappan burials discovered in Rakhigarhi cemetery.

Burial Type		Features	RGR 7.2 (A1 / A2)	RGR 7.3 (B1 / B2)
Primary Burial (n = 41)	Typical (n = 34)	Singular Supine inside Simple Pit	BR 01, 02, 03, 04, 07 08, 09, 10A,10B, 11,12, 13,14,15,17A,17B, 18A, 18B, 20A,20B,20C,23, 24, 25, 26, 27, 28, 29, 35, 36	BR 01B, 03A, 03B, 04
	Atypical (n = 7)	Brick-lined Grave	BR 19, 22, 31, 33	Nil
		Prone-Positioned	BR 33	BR 01A, 02A1
		Multiple	Nil	BR 02A1, 02C1
Secondary Burial (n = 5)		Single	BR 21, 32B	Nil
		Multiple	Nil	BR 02A2, 02B,02C2
Symbolic Burial (n = 6)		Small pit (circular or square)	BR 06, 16, 34	Nil
		Pit Chamber	BR 05, 32A	BR 05
Unused Pit Chamber (n = 1)		Brick-lined Empty Chamber	BR 30	Nil

*N/A: Not Available.

The votive pot numbers found in each burial group are depicted in a box plot (Fig 8B). The difference in pot numbers between the primary typical and secondary burials was statistically insignificant (Wilcoxon rank sum test: W = 66.5, p-value = 0.2341). However, we found that the pot numbers for symbolic burials were significantly higher than those for primary typical graves (Wilcoxon rank sum test: W = 158, p-value = 0.007606).

Unlike the cases of symbolic burials, nothing was discovered inside A2/BR30: no human or animal bones, and no grave goods. We suspected that A2/BR30 might have been built in preparation for a funeral but was eventually abandoned for reasons as yet unknown. If this actually is as we conjecture, an unused pit chamber, it is a very rare case in the field of Harappan archaeology. Only one similar case, from Farmana cemetery, previously has been reported [10].

### Skeletons

Among the various graves excavated in Rakhigarhi cemetery, human remains were found only in primary and secondary burials, not in any presumptive symbolic or unused pit chambers. Well-preserved bones were found, as is typical, mainly in primary graves; skeletons were found also in cases of secondary burial, though their conservation status was generally poor.

Overall, our excavation at Rakhigarhi cemetery revealed at least 46 sets of complete or partial skeletal remains. Of them, 41 (89.1%) were discovered in primary burials, and 5 (10.9%) in secondary burials. In the primary burials, though the individuals were generally placed in supine positions, prone-positioned individuals were also found in a few exceptional cases (A2/BR33, B1/BR01A and B2/BR02A1).

After excluding the cases of only fragmentary or incomplete skeletal remains, only 37 individuals were finally subjected to anthropological examination. Overall, there were 9 individuals with more than half of the skull preserved; in 14 individuals meanwhile, the pelvic bones remained. In the age estimation, we found 8 subadults (under 18 years old) and 17 adults; fully 12 cases were indeterminate due to skeletal incompleteness or poor preservation. Among the 17 adults, 5 seem to have died at young age, 11 at middle age, and only one at old age (Table 2). We also sub-divided the age at death of the children. Two children (A2/BR10A and A2/BR17A) seem to have died at 2–4 years and one child (A1/BR03) at 3–5 years. For A2/BR20B, though the skeleton was judged to be that of a child, the age could not be estimated (Table 2).

**Table 2:** Anthropological profile of the skeletons from Rakhigarhi cemetery.

Sex / Age	Child	Adolescent	Adult			Indeterminate	Total
			Young	Middle	Old		
Male	0	2	2	2	0	1	7
Female	0	0	3	4	1	2	10
Indeterminate	4	2	0	5	0	9	20
Total	4	4	5	11	1	12	**37**

As for the individuals’ sex, we estimated that there were 7 males and 10 females. For all of the children (n = 4), some of the adolescents (n = 2) and adults (n = 4) and most of the age-indeterminate individuals (n = 10), we could not estimate the sexes. In the light of the anthropological information obtained, we tried to interpret the archaeological data collected from Rakhigarhi cemetery. The data are summarized in Table 3.

**Table 3:** The archaeological and anthropological details of the burials in Rakhigarhi cemetery.

Locality	Trench	Burial No.		Group	Locus in Trench	Gender/Age	Burial Type / Position of the Dead	Votive Pots inside Grave	Ornaments	Other Remarks
RGR 7.2 (n = 42)	**A1** (n = 6)	**BR 01**		III	SE	Male / 36–50 yrs	Primary / Supine	7		
		**BR 02**		II	SE	Male / 16–18 yrs	Primary / Supine	6		Good Preservation of Skeletal Remains
		**BR 03**		III	NE	Indeterminate /3-5 yrs	Primary / Supine	2		Shallow Grave
		**BR 04**		III	NE	Female / 36–50 yrs	Primary / Supine	4	Bracelet (1 shell on left arm)	
		**BR 05**		III	NE		Symbolic / Nil	**27**		
		**BR 06**		III	SW		Symbolic / Nil	6		
	**A2** (n = 36)	**BR 07**		III	SE	Indeterminate /36-50 yrs	Primary / Indeterminate	22		Encroachment 07 by 08 and 09
		**BR 08**				Indeterminate /Adult	Primary / Indeterminate	7		
		**BR 09**				Indeterminate /36-50 yrs	Primary / Supine	7		
		**BR 10**	**A**	III	SE	Indeterminate /2-4 yrs	Primary / Supine	3		Encroachment B by A
			**B**			Indeterminate /Adult	Primary / Supine	2		
		**BR 11**		III	SE		Primary / Indeterminate	12		Disturbed during Early Historic
		**BR 12**		III	SE	Indeterminate /Adult	Primary / Supine	11	Anklet (steatite micro-beads)	
		**BR 13**		III	NE	Indeterminate /Adult	Primary / Indeterminate	7		Encroachment 14 by 13 and 15 Both 13 and 15 have shell spoon inside small pot
		**BR 14**					Primary / Indeterminate	13		
		**BR 15**				Female /Indeterminate	Primary / Supine	8		
		**BR 16**		I	SE		Symbolic / Nil	13		
		**BR 17**	**A**	II	SE	Indeterminate /2-4 yrs	Primary / Supine	3		Relation of A and B is not certain
			**B**				Primary / Indeterminate	6		
		**BR 18**	**A**	III	SW	Indeterminate /Adult	Primary / Indeterminate	8		Encroachment B by A
			**B**			Indeterminate /Adult	Primary / Indeterminate	6		
		**BR 19**		II	SW	Female / 21–35 yrs	Primary / Supine	16	Bracelet (1 shell on left arm) Necklace (2 Carnelian beads)	Brick-lined Grave
		**BR 20**	**A**	III	NE	Indeterminate /36-50 yrs	Primary / Supine	2		Encroachment A by B, B by C
			**B**			Indeterminate /Child	Primary / Indeterminate	3		
			**C**				Primary / Indeterminate	2		
		**BR 21**		III	NE		Secondary /few bones	17		
		**BR 22**		II	SE	Female / 21–35 yrs	Primary / Supine	9	Bracelets (2 coppers:left and right arm) Terracotta Ring (right chest)	Most Elaborate Grave Structure (Brick-lined)
		**BR 23**		III	SE	Indeterminate /Adolescent	Primary / Supine	Indeterminate	Ring (copper on left hand)	
		**BR 24**		III	SE	Female /Adult	Primary / Supine	2		
		**BR 25**		III	SE	Male / 36–50 yrs	Primary / Supine	5		
		**BR 26**		III	SW	Indeterminate /36-50 yrs	Primary / Supine	13		
		**BR 27**		III	SE		Primary / Indeterminate	4		Disturbed during Early Historic
		**BR 28**		III	SE	Indeterminate /Adult	Primary / Supine	6		
		**BR 29**		III	SE		Primary / Indeterminate	12		Encroachment by BR 25
		**BR 30**		II	SW		Unused	0		
		**BR 31**		II	SW	Female / >50 yrs	Primary / Supine	22	Necklace (1 stone / 1 Faience bead) Bracelet (1 shell on right arm) Anklet (steatite micro-beads)	Brick-lined Grave
		**BR 32**	**A**	III	SW		Symbolic / Nil	18		Encroachment A by B
			**B**				Secondary / Nil	3		
		**BR 33**		II	SW	Female /21-35 yrs	Primary / **Prone**	**34**		Brick-lined Grave
		**BR 34**		II	SW		Symbolic / Nil	**27**		
		**BR 35**		III	SW	Indeterminate /12-16 yrs	Primary / Supine	10	Bracelet (4 shells–left arm / 2 coppers and 12 stone beads–right) Necklace (beads of different size and materials)	
		**BR 36**		I	SE	Female /36-50 yrs	Primary / Supine	7	Bracelets (2 shells on left arm)	Good Preservation of Skeletal Remains
RGR 7.3 (n = 11)	**B1** (n = 3)	**BR 01**	**A**	III	N	Male /21-35 yrs	Primary / **Prone**	**46**		A-Good Preservation of Skeletal Remains Relation of A and B is not certain
			**B**				Primary / Indeterminate	Indeterminate		
		**BR05**		III	S		Symbolic / Nil	20		
	**B2** (n = 8)	**BR02**	**A1**	III	N	Male /21-35 yrs	Primary **/ Prone**	**37**		Well-plastering for A1 Both A1 and C1 have small pot under knee A1 and C1—Good Preservation of Skeletal Remains
			**A2**			Indeterminate /36-50 yrs	Secondary / Nil			
			**B**			Female /36-50 yrs	Secondary / Nil			
			**C1**			Male /16-18 yrs	Primary / Supine			
			**C2**			Indeterminate /Indeterminate	Secondary / Nil			
		**BR 03**	**A**	III	**S**	Male /Indeterminate	Primary / Supine	31		Encroachment of B inside A
			**B**			Indeterminate /Indeterminate	Primary / Supine	Indeterminate		
		**BR 04**		III	**S**	Female /36-50 yrs	Primary / Supine	15	Bracelets (2 copper: 1 left and right)	

### Graves for subadults

**Fig 9:**
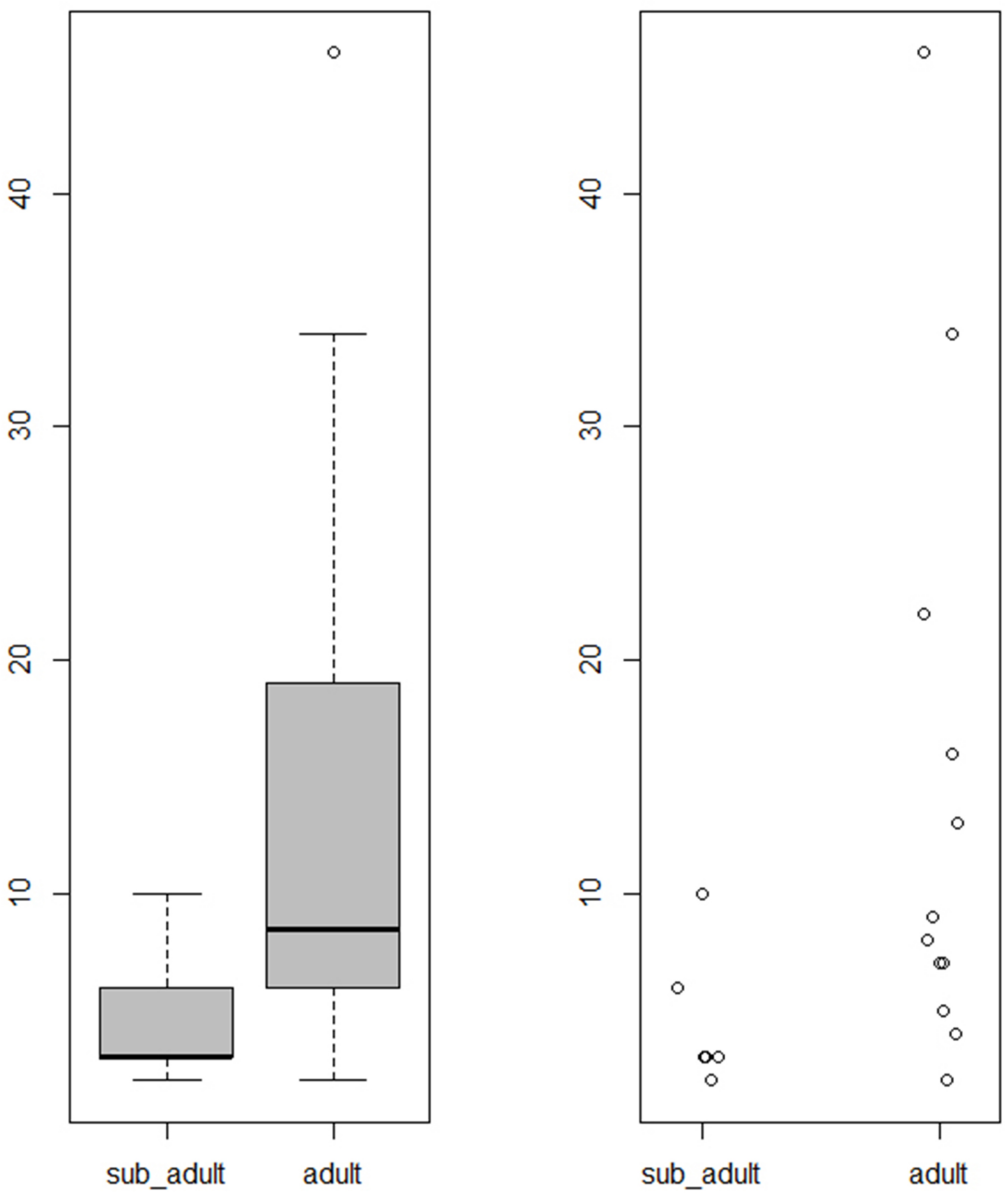
Box and scatter plots of votive pot numbers found in subadult and adult graves.

When we depicted the votive pot numbers of subadult (under 18) and adult graves, the former's burials included significantly fewer votive pots than did the latter (Fig 9). The difference between them was statistically significant by Wilcoxon rank sum test (W = 58.5, p-value = 0.03874). In general, according to particular cultures, subadults’ deaths are dealt with quite differently. Some cultures did not make graves for their dead children at all, while others constructed children’s graves as good as or even better than adults' [54]. As fewer votive pots were found in the subadults’ burials than in the adult graves, the Rakhigarhi people might have treated their children's deaths in a somewhat different way from adults’.

### Graves for women

**Fig 10:**
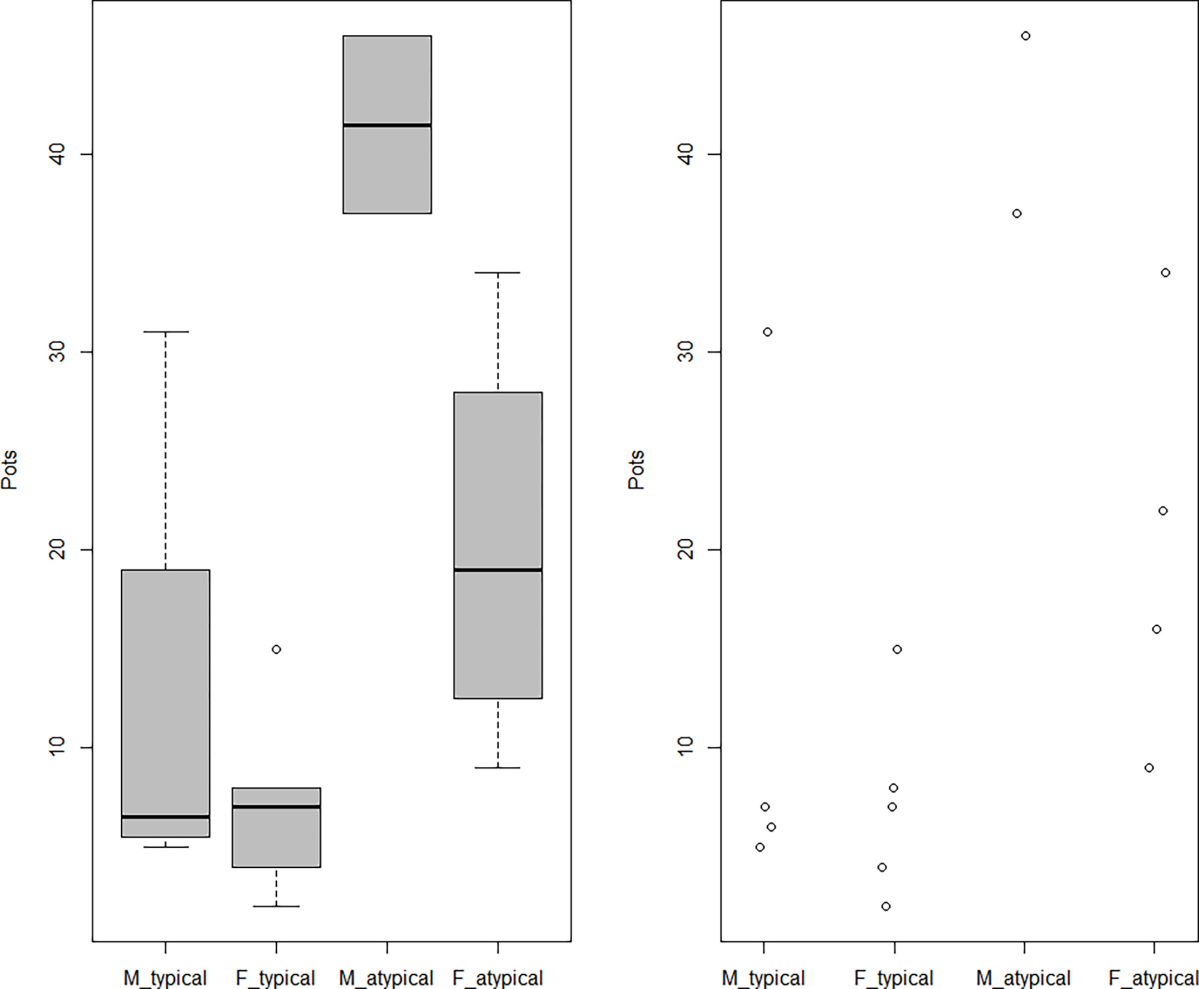
Box and scatter plots of votive pot numbers in typical and atypical graves according to sex. M_ and F_ means male and female, respectively.

The votive pot number found in atypical graves was higher than in typical graves (Fig 8A). As the number of votive pots in those graves somewhat differed by sex (higher in males’ graves than females’) (Fig 10), there might have been, among some Rakhigarhi people at least, discriminatory attitudes toward women with respect to the construction of graves. In a statistical analysis of the votive pots from atypical burials, however, we failed to find any significance for difference by sex (Pooled variance t-test, t = -2.5266, df = 4, p-value = 0.0649), possibly due to the insufficient sample size. Our estimates will be firmer as reports from similar cases become available.

### Multiple individuals inside the pit

**Fig 11:**
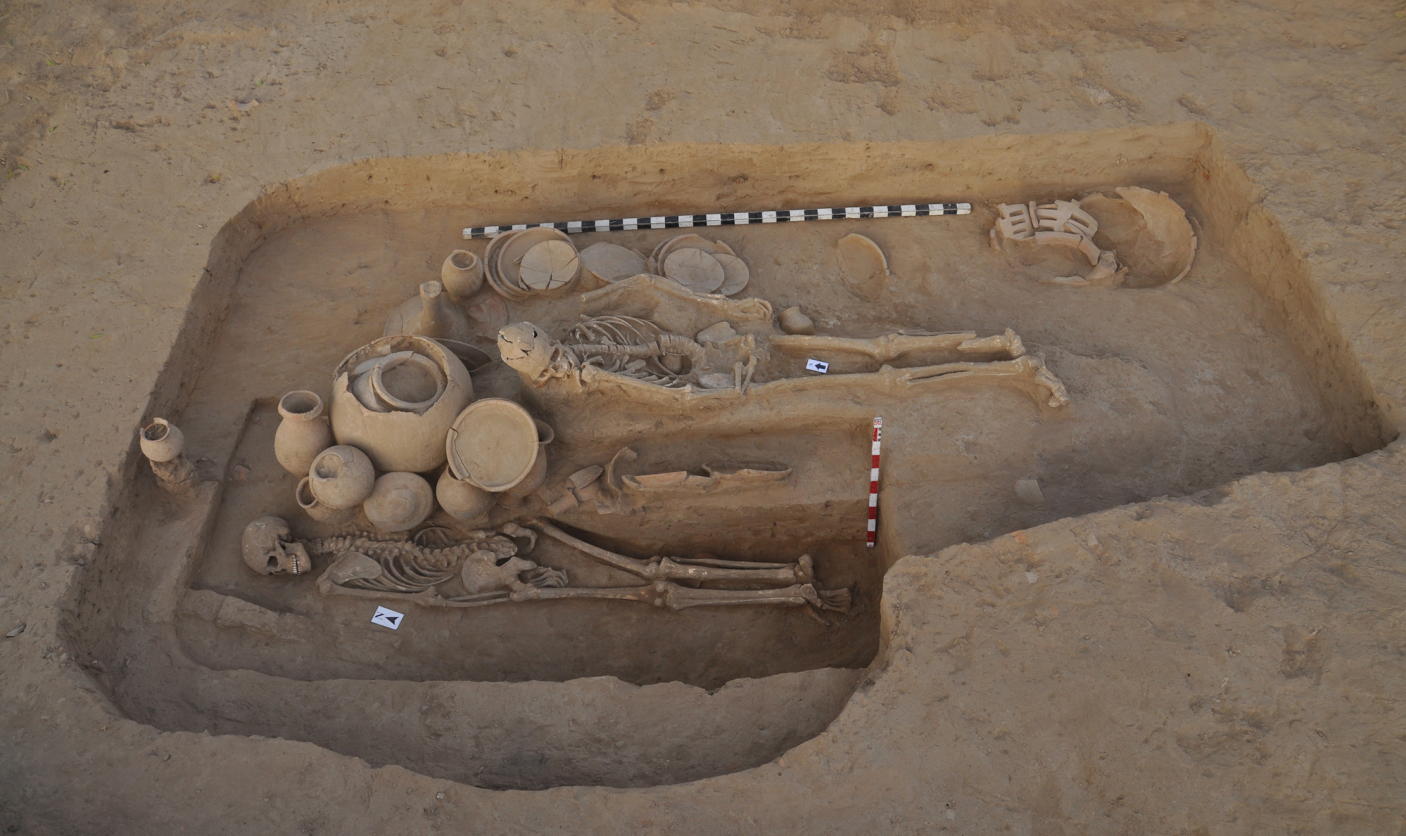
Five individuals buried together inside the same pit. A prone-positioned male (B2/BR 02A1) and a supine-positioned male (B2/BR 02C1) were found together.

While the great majority of interments in Rakhigarhi cemetery contained only one individual, interestingly enough, five individuals (A1/A2/B/C1/C2 of B2/BR02) were found to be have been placed together inside the same pit. According to the archaeological context, we conjecture that all of those individuals had been buried together at the same time. Among them, B2/BR A1 and C1 were primary burials, whereas B2/BR A2, B and C2 were secondary. While only bone fragments were found inside the secondary burials, the skeletons discovered in primary burials showed an excellent preservation status. The skeletons from the primary burials were determined to be males; the age estimations were 21–35 yrs for B2/BR A1 and 16–18 yrs for C1 (Fig 11). In this multiple-individual grave, the number of grave goods was far numerous than in any of the other primary graves; moreover, various types of bowls rarely found in other burials were discovered here.

In two primary burials (B2/BR A1 and C1), we found the same kind of small pot positioned in the same way under the knees (S4 Fig). In Rakhigarhi cemetery, there were two other graves very similar to B2/BR A1 and C1. In the A2/BR13 and 15 burials, we found that a similarly shaped shell spoon had been placed in the same way inside the small pots (S5 Fig). The manner of arrangement of grave goods is very important, as it sometimes suggests that the two individuals buried together had a close relationship in life. Even so, as there have been very few reported parallels in the Harappan funerary context, this kind of burial remains enigmatic to us.

### Ornaments of the buried bodies

**Fig 12:**
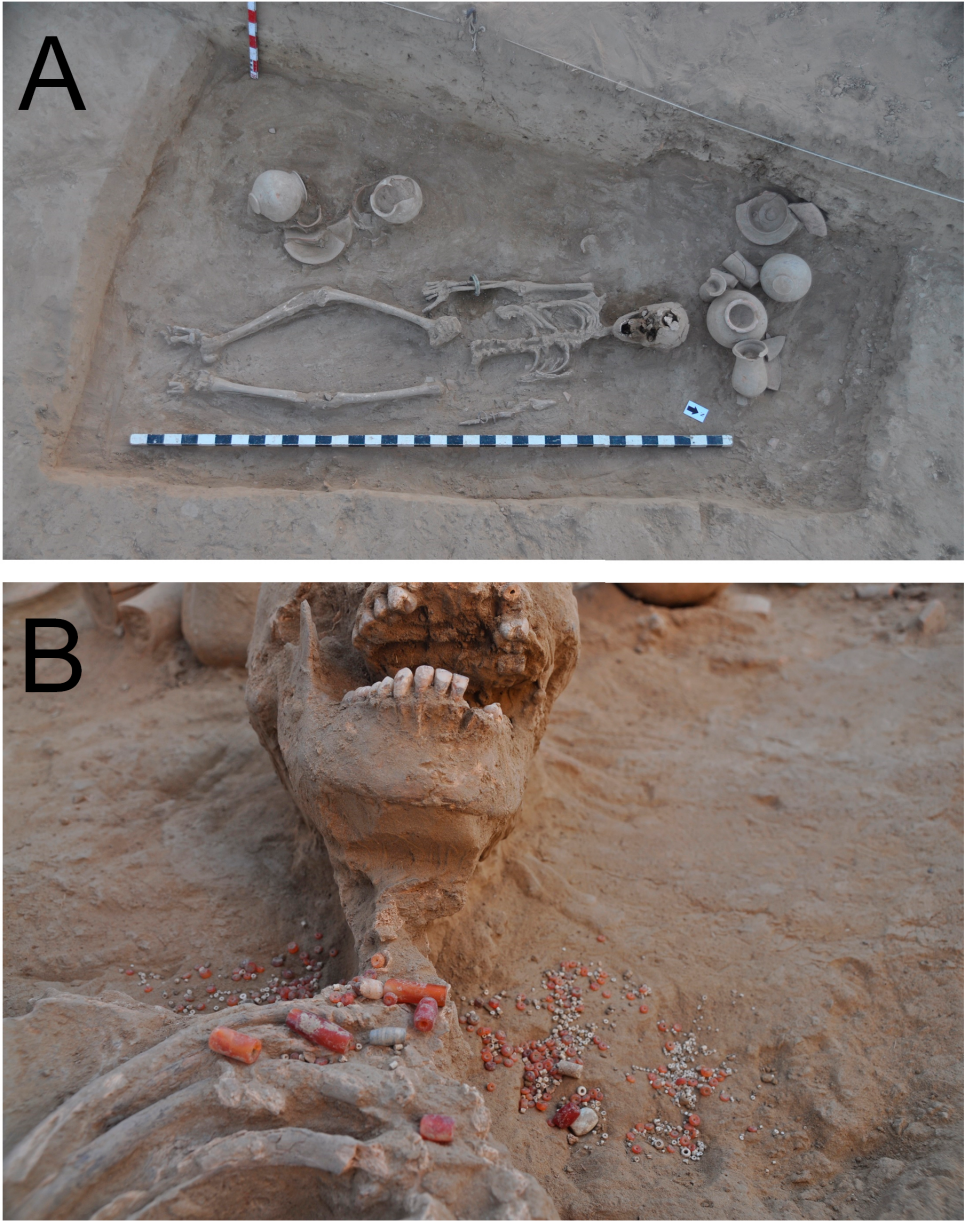
(A) Young individual (A2/BR35) discovered wearing necklaces and bangles. (B) The necklace worn by the A2/BR35 individual.

Among the anthropologists’ sex-determined cases, we found that only the females (n = 7) wore bangles. These ornaments were also found in burial A2/BR35, for which we discovered a young individual (12–16 years old, sex not determined) wearing necklaces and bangles made of copper, shell and gemstones (Fig 12A and 12B). Initially, we conjectured that this individual might have been of a high social class. However, this hypothesis had to be abandoned later, as the grave architecture of A2/BR35 was too humble to be comparable to other, elaborate graves found in Rakhigarhi cemetery. This case is a good example of how care must be exercised when making social-status determinations for Harappan burials based on only a limited number and/or variety of artifacts.

### Brick-lined graves

**Fig 13:**
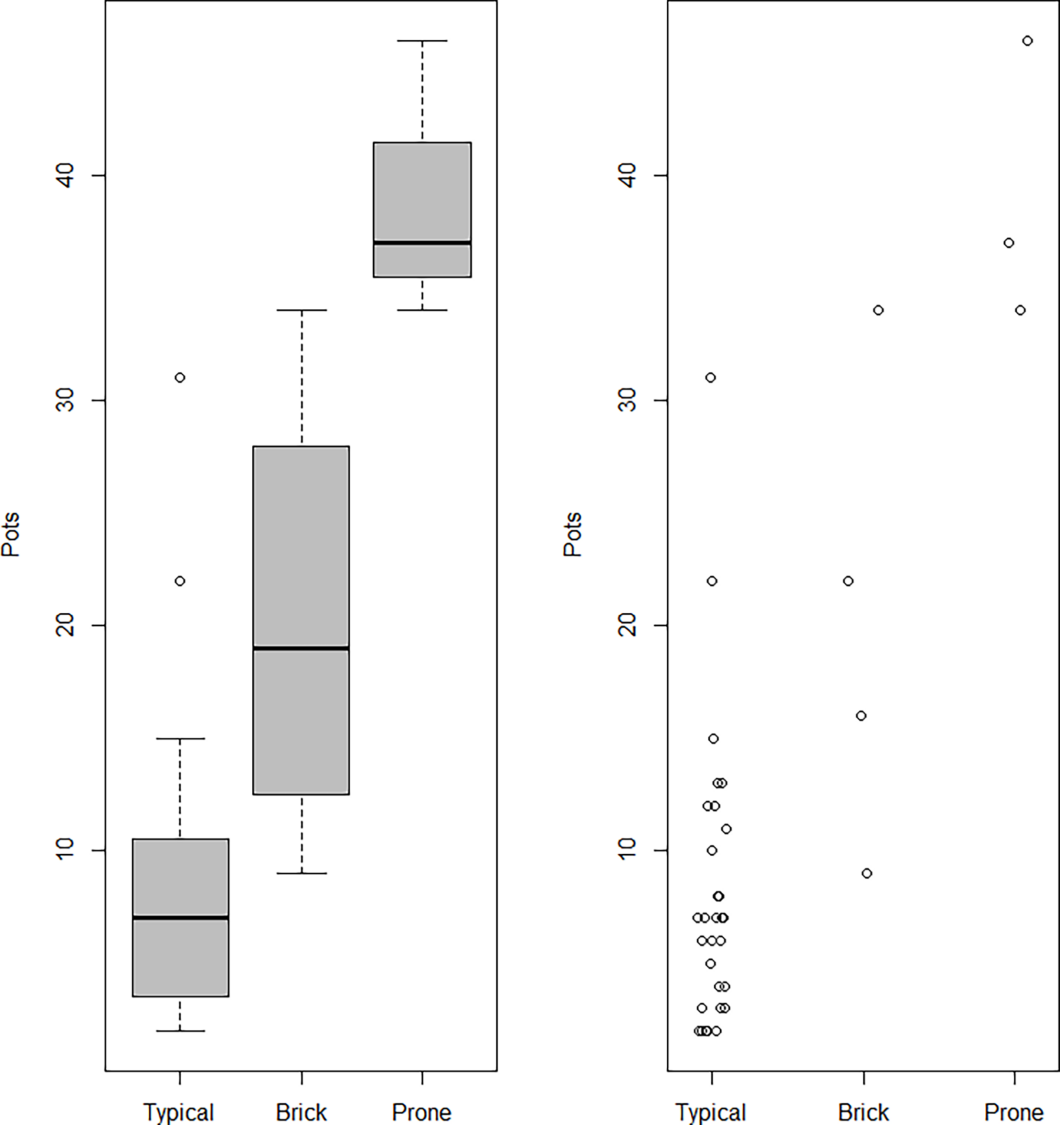
Box and scatter plots depicting votive pot numbers from primary typical, brick-lined and prone-position graves.

Actually, exotic items, such as inscribed seals or ritual objects (e.g. terracotta Mother goddesses) have never been found in any Harappan-period graves, not even in elaborate ones [7,8,10,11]. Likewise, the burial structures and grave goods of the Rakhigarhi necropolis have all been determined to be generally humble in nature. It was easy for us to conjecture as to a common pattern among primary typical burials in the cemetery: for example, only one individual was interred supinely in a plain grave. Even so, when we come to the details of each burial, differences possibly reflective of ritual status and/or the dynamic situation prevailing at the time of the individual’s death seem to have determined grave structures or offering goods discovered in various burial cases. Among the atypical primary interments, we noted brick-lined graves (A2/BR19, 22, 31, and 33) as an example of such unique burials found in Rakhigarhi cemetery. Our box and scatter plots show that the brick-lined graves included more votive pots than did typical interments. This difference was found to be statistically significant (Wilcoxon rank sum test, W = 111.5, p-value = 0.01074) (Fig 13).

The brick-lined graves showed the following features.

**A2/BR19:** A rich variety of grave goods was identified. We discovered the skeletons of a young woman (21–35 years old). The bricks had been crushed into small pieces and mixed with lime for strengthening. The brick-lined wall was found only at the head of the buried individual.

**A2/BR22:** This grave was made with great care. A young woman (21–35 years old) was found inside. The individual wore copper bangles on both arms. A brick-lined wall was confirmed to be present near the head of the individual.

**Fig 14:**
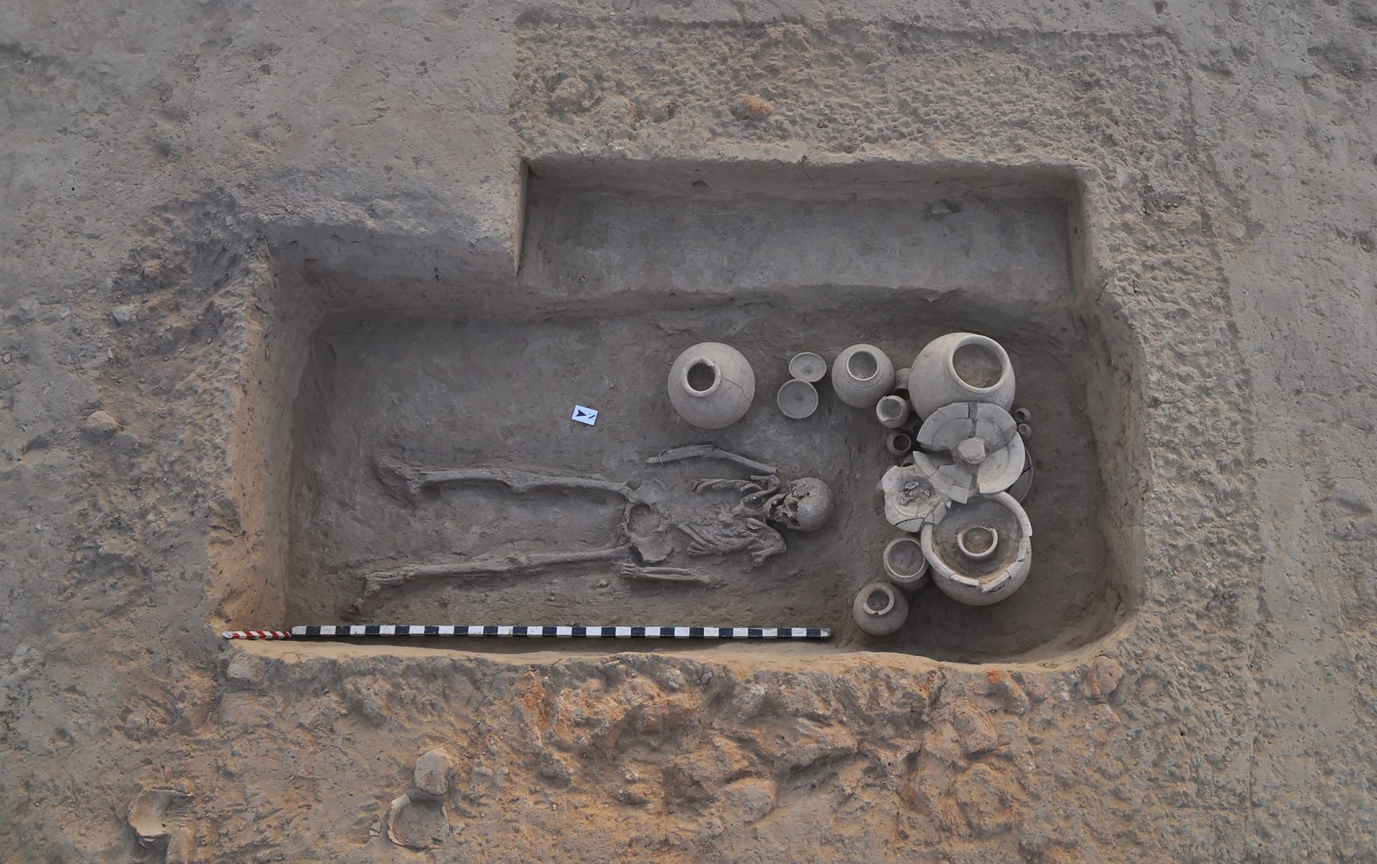
Brick-lined burial at Rakhigarhi cemetery. A large quantity of grave goods was found inside A2/BR31.

**A2/BR31:** The burial wall was made with a mixture of burnt bricks and lime. A large number of pots was found inside the grave. The individual was estimated to be an old female (> 50 years) (Fig 14).

**A2/BR33**: Bricks were found in the burial wall. A large amount of pottery was found inside. The individual was a female (21–35 years old).

Brick-lined graves also have been reported for other Harappan cemeteries (i.e., Harappa, Kalibangan, Farmana, and Lothal) [7]. These burials were assumed to have been for important men and to be representative of the intra-variability of social and ritual status, due to their impressive and unique elaborateness [7,8]. Correspondingly, at Rakhigarhi cemetery, the brick-lined burials were among the most elaborately constructed graves, implying a high social or ritual status for the deceased. Notwithstanding the many similarities, there were also some differences between the brick-lined burials in Rakhigarhi and similar graves of other Harappan cemeteries. While the former used a mixture of crushed burnt bricks and lime, the latter were made mainly of mud bricks; and whereas the Rakhigarhi graves built the brick-lined wall only near the head of the individuals, the other, similar cases at other cemeteries had walls constructed that completely surrounded the graves.

We note that every individual discovered in a brick-lined burial was likely, by anthropological examination, to have been female. Therefore, if we accept the hypothesis that the people buried in the brick-lined graves actually belonged to the dominant group in Rakhigarhi society, we must reconsider the social role of some Harappan females at that time.

### Prone-positioned individuals

Although approximately 600 prone-positioned cases have been reported from archaeological sites over the past several decades [55], such individuals remain very rare. Then, what was prone-positioning for? It has been thought that, traditionally, prone-positioned burials represented a way to treat the bodies of individuals who had been shamans (or witches), disabled individuals, and/or those who had been ostracized for any reason (e.g., criminality, religious nonconformity) from the community [56–59]. Most prone-positioned individuals have been revealed to be males, and the grave goods found inside such graves have been very few [55].

In our study, we found prone-positioned individuals in some of the graves of Rakhigarhi cemetery (A2/BR33, B1/BR01A and B2/BR02A1). Details on each burial are summarized as follows.

**Fig 15:**
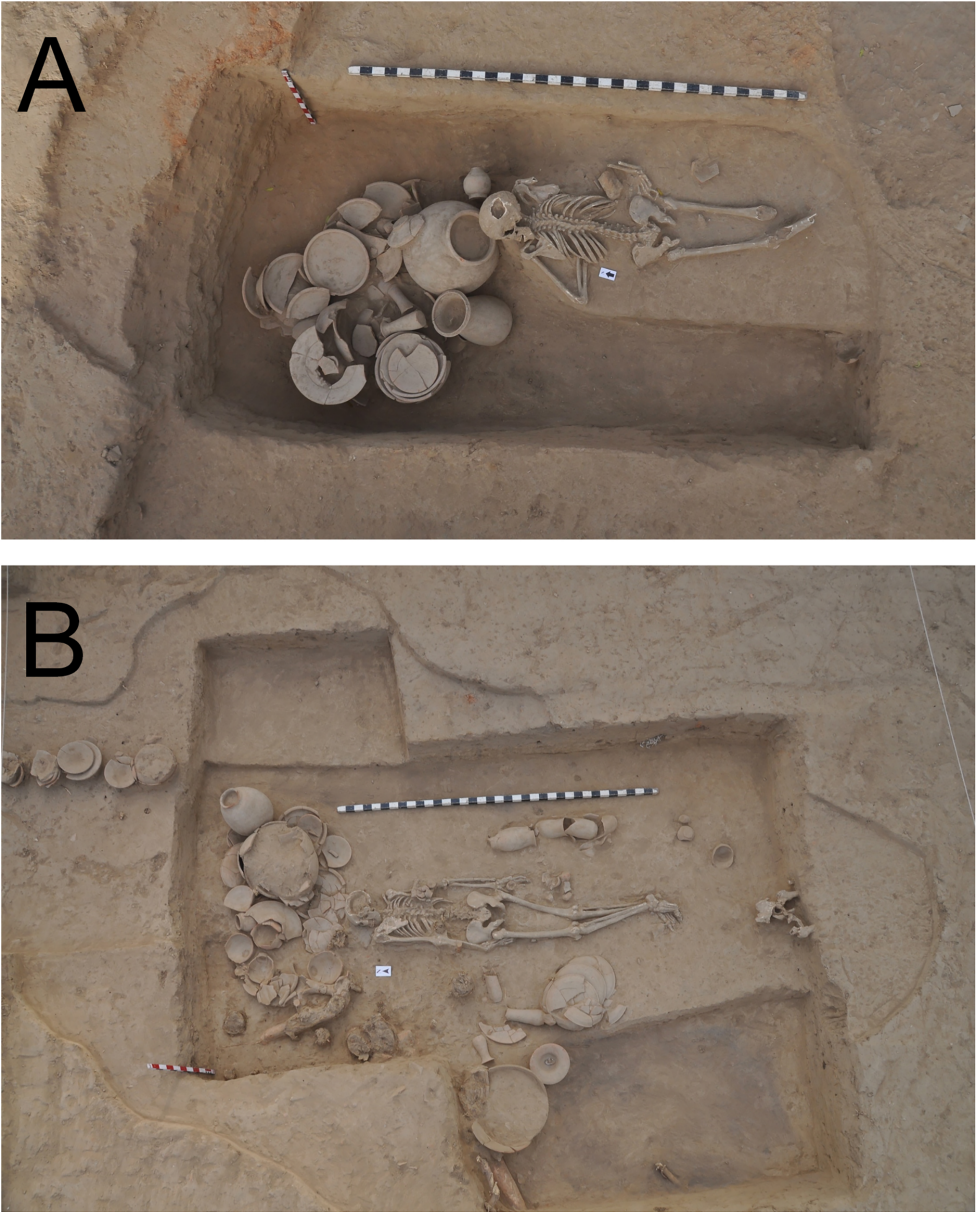
Prone-positioned individuals in (A) A2/BR33 and (B) B1/BR 01A.

**A2/BR33:** A brick-lined, atypical burial. A large amount of pottery was found inside the grave. The individual was a female (21–35 yrs.). She was buried in a prone position *while looking left* (Fig 15A).

**B1/BR01A:** Traces of funeral rituals (burnt ashes, animal bones, a large jar) were identified inside the grave at the northeast. Fine silt soil had been piled up as if for a makeshift bed. The young adult male (aged 21–35 yrs.) was in the prone position, *facing to the left side*. The quantitative and qualitative features of the votive pottery (Fig 15B) were more remarkable than in typical interments.

**B2/BR02A1:** A male (aged 21–35 yrs.) was found prone-positioned and *facing to his left side*. The disposers had arranged large numbers of votive pots inside the grave (Fig 11). Next to him, upon a higher elevation, a male individual (B2/BR 02C1, aged 16–18 yrs.) also lay in the supine position. We wondered why the two individuals had been placed in different positions. Arcini [55] speculated that the prone position might have symbolized a submissive posture. If this was the case, the prone-positioned B2/BR 02A1 individual might have been arranged in such a way as to pay homage to the supine-positioned C1 individual. Although this seems a strong hypothesis, we will need to reconsider the present case, particularly because in the same Rakhigarhi cemetery, there were other prone-positioned individuals (A2/BR 33 and B1/BR 01A; Fig 15) who had been buried alone, in the absence of higher status individuals.

In Rakhigarhi cemetery, what stands out in prone-position graves is that the individuals appear unlikely to have been social deviants. We could not find any evidence of physical restraint such as intentional bending at the knees and/or tying of the feet to the buttocks. Neither were there any signs that any of the individuals had been social deviants. It is also interesting that among the Rakhigarhi individuals that were found lying in prone position, some were not, as is typical around the world, facing down [55–59] (so as to block their view of the sky and indeed to prevent their ever breathing again), but rather looking to the left.

We conjecture that those prone-positioned individuals might actually have belonged to the upper classes of Rakhigarhi society, principally because they were given elaborate burials and interred with a large number of grave goods. Our box and scatter plots clearly depict that the votive pot quantity in the prone-positioned burials is significantly greater than in the primary interments of the same cemetery (Wilcoxon rank sum test, W = 93, p-value = 0.005024) (Fig 13). However, we admit that in order for this hypothesis to be generally accepted, more research into similar cases from other Harappan cemeteries is required.

### Burial on bed of pottery

We finally comment on the presence of burials on beds of pottery. In Rakhigarhi cemetery, we found graves (A2/BR33 and B2/BR02C1) wherein the soil had been built up with pots like a bed upon which the body was laid. Considering the grave architecture and amounts of grave goods inside them, burials A2/BR 33 and B2/BR 02C1 seem to have been of high-ranking people in Rakhigarhi society.

A similar burial on bed of pottery was reported for another Harappan grave. In Kalibangan cemetery, the prone-positioned individual of No. 29 grave had been laid down on stacked votive pots [8]. This report did not attract much attention at the time of the report. However, we noticed that this Kalibangan No.29 burial (S6 Fig) shared many features (grave structure, votive pots, and prone-positioned posture) with our Rakhigarhi grave A2/BR33 (Fig 15A). The similar finding was also reported at Harappa, the type site of the Harappan Civilization [7]. A recent report from a 7^th^ century Anglo-Saxon cemetery is also suggestive to us, because it indicates that a real bed had been put in the grave, not a platform made of soil and pots, and that the individual had been a high-ranking female [60]. Although the discovery of similar burials has been very rare to date, we could not rule out the possibility that such a funeral custom might have been followed over a much wider area than we had initially considered.

## Conclusion

In principle, there was always a high probability and expectation that cemeteries would be discovered in the vicinity of Harappan cities or towns. However, with respect to Harappan megacity sites, Harappan cemeteries have not been reported in sufficient numbers to date. The lack of a cemetery within the Mohenjo-daro area, for example, represented a serious inconsistency between the archaeological data and the literature. The only relevant discoveries there were a disorganized scattering of 43 skeletal remains within the city district [18] and a few isolated graves and skeletons at construction sites outside of the city [7]. Ganweriwala, another large Harappan city, has not been properly excavated yet, as it is situated in a volatile area near the India–Pakistan border. A large cemetery area was identified at the Dholavira site, but only a few graves have been excavated so far [61].

Of the five megacities of the Harappan Civilization, an actual cemetery district (area: *ca*. 0.8–1.2 ha) has been discovered only at the Harappa site. Several excavations in recent years have provided extensive data on approximately 280 burials [7]. In the cemetery (R-37) at Harappa, archaeologists have found many unique cases [7] that were matched in our current report on Rakhigarkhi cemetery. Reports on cemeteries have also been made from the smaller Harappan towns of Lothal [62], Kalibangan [8], and Farmana [9]. While this archaeological data, in sum, still falls short of a comprehensive accounting of Harappan cemeteries, we can summarize it as follows.

In brief, Harappan-period cemeteries were generally built on the periphery of residential settlements. Most burials included only one individual. The body was fully extended in the supine position, with the head to the north. A number of votive pots were placed in the graves at the head end. While some graves had no or few pottery, certain burials included various kinds of pots. Overall, people over vast areas covering the Northwestern parts of South Asia might have shared common burial practices and heritages during the Mature Harappan period.

Like the other cemetery sites in the Ghaggar Basin, Rakhigarhi cemetery is representative of the Mature Harappan period, date-estimated to 2,500–2,000 BCE. By our three-year survey, we obtained scientific information from the graves of the cemetery. We found that various types of graves co-existed in different proportions. Primary interments were identified most commonly in the cemetery, followed by secondary, symbolic, and unused (empty) graves. There were significant differences in mortuary rituals especially between primary typical and atypical graves. Prone-positioned individuals are another noteworthy finding for Rakhigarhi cemetery, because we need to reconsider the validity of the common pre-conception about prone-positioned burials in archaeology, at least as far as the Harappan Civilization is concerned.

In this study, systematic analysis of Rakhigarhi cemetery was successfully achieved by close collaboration between archaeologists and anthropologists. Although the general patterns of burial and mortuary practice at the Rakhigarhi necropolis look similar to those of other Harappan cemeteries, there was also much concrete information acquired that is unique to the present investigation. All in all, the current report provides a rare glimpse into the Harappan people’s practices and rituals relating to burial of their dead. But more work remains to be done.

## Supporting information

S1 Fig Examples of naming of burial pits at RGR 7.2/A2. (JPG)

S2 Fig Investigations ongoing at Rakhigarhi cemetery. (JPG)

S3 Fig Secondary burial (A2/BR 21) at Rakhigarhi cemetery. The pot burial was placed in a circular pit. Adult human skull and a few long bones were kept inside a jar. The skeletons might have been buried temporarily in one place before finally being moved for a pot burial. Note the animal bones placed on the dish.
(JPG)

S4 Fig Same kind of small pots was found in the same way under individuals’ knees at two different primary burials: (A) B2/BR A1 and (B) B2/BR C1.
(JPG)

S5 Fig Pottery set for one individual’s grave was similar to those of two adjacent burials: (A) A2/BR13 and (B) A2/BR 15. The shell spoons were found inside the small pots.
(JPG)

S6 Fig Drawing of burial No. 29 found in Kalibangan cemetery. The grave structure of this burial is very similar to our Rakhigahi A2/BR33 case. The figure is here redrawn from the original of the previous report [8].
(JPG)
