# Supplementary figures and images for: Archaeological and anthropological studies on the Harappan cemetery of Rakhigarhi, India
Source: PLoS One. 2018 Feb 21;13(2):e0192299. doi: 10.1371/journal.pone.0192299 (PMC5821334; doi:10.1371/journal.pone.0192299)

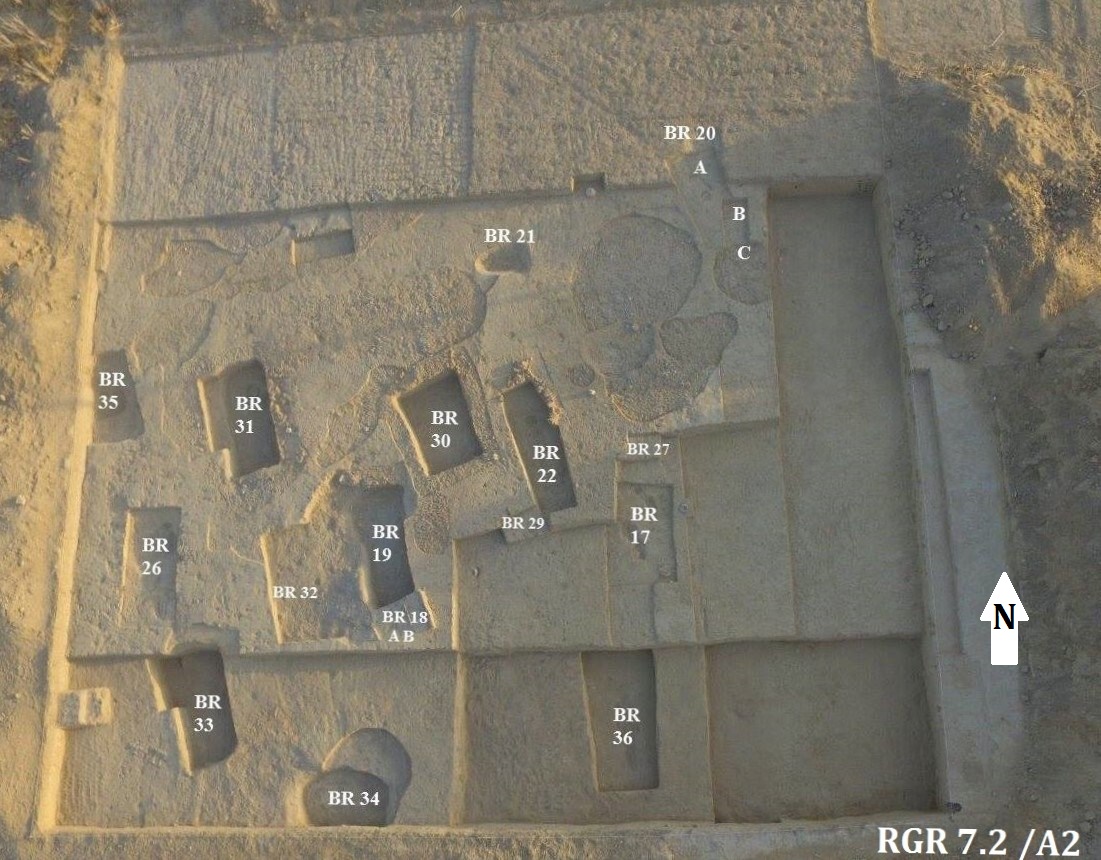

Supplement: S1 Fig — (JPG) [file pone.0192299.s001.jpg]

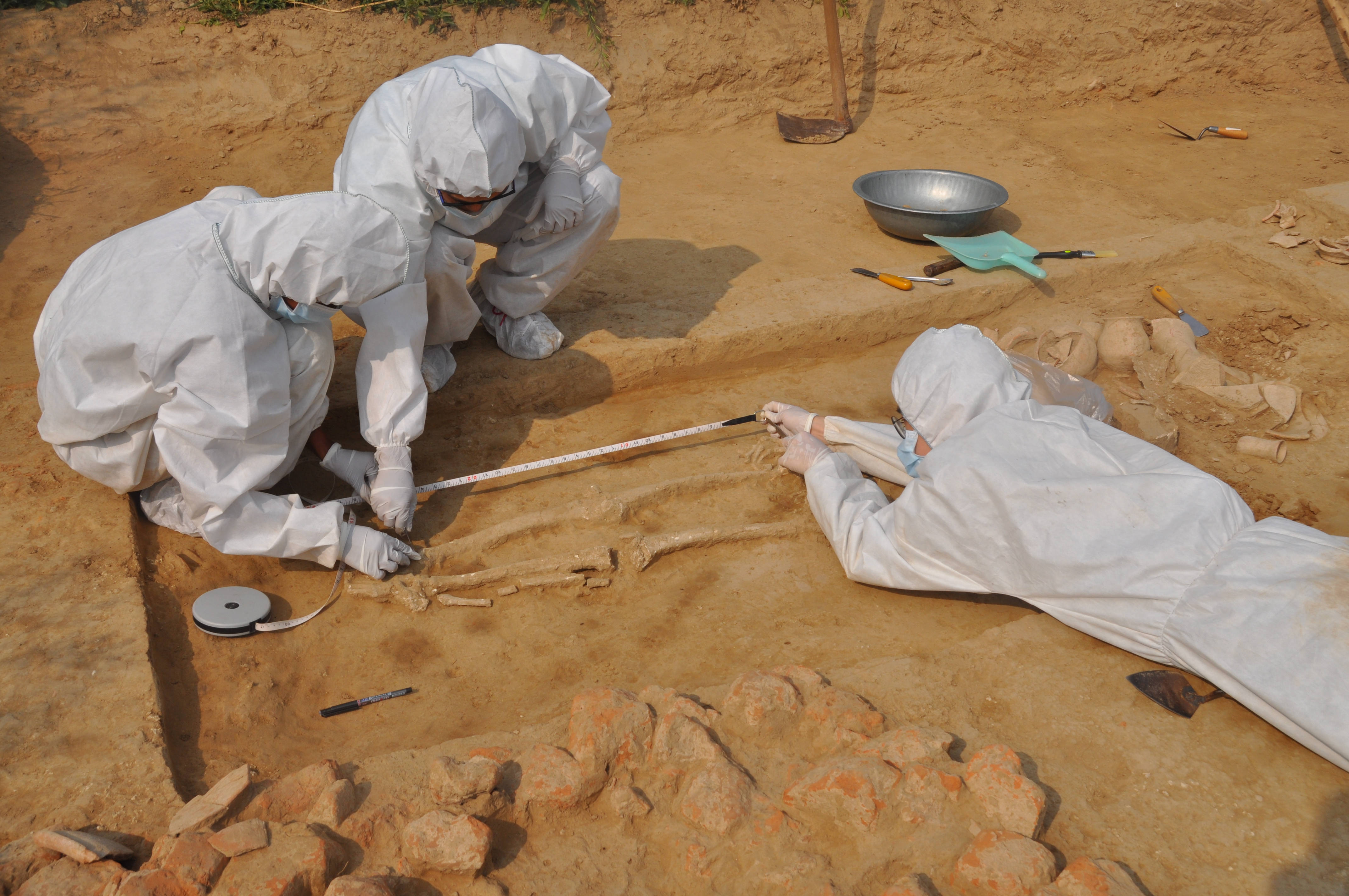

Supplement: S2 Fig — (JPG) [file pone.0192299.s002.JPG]

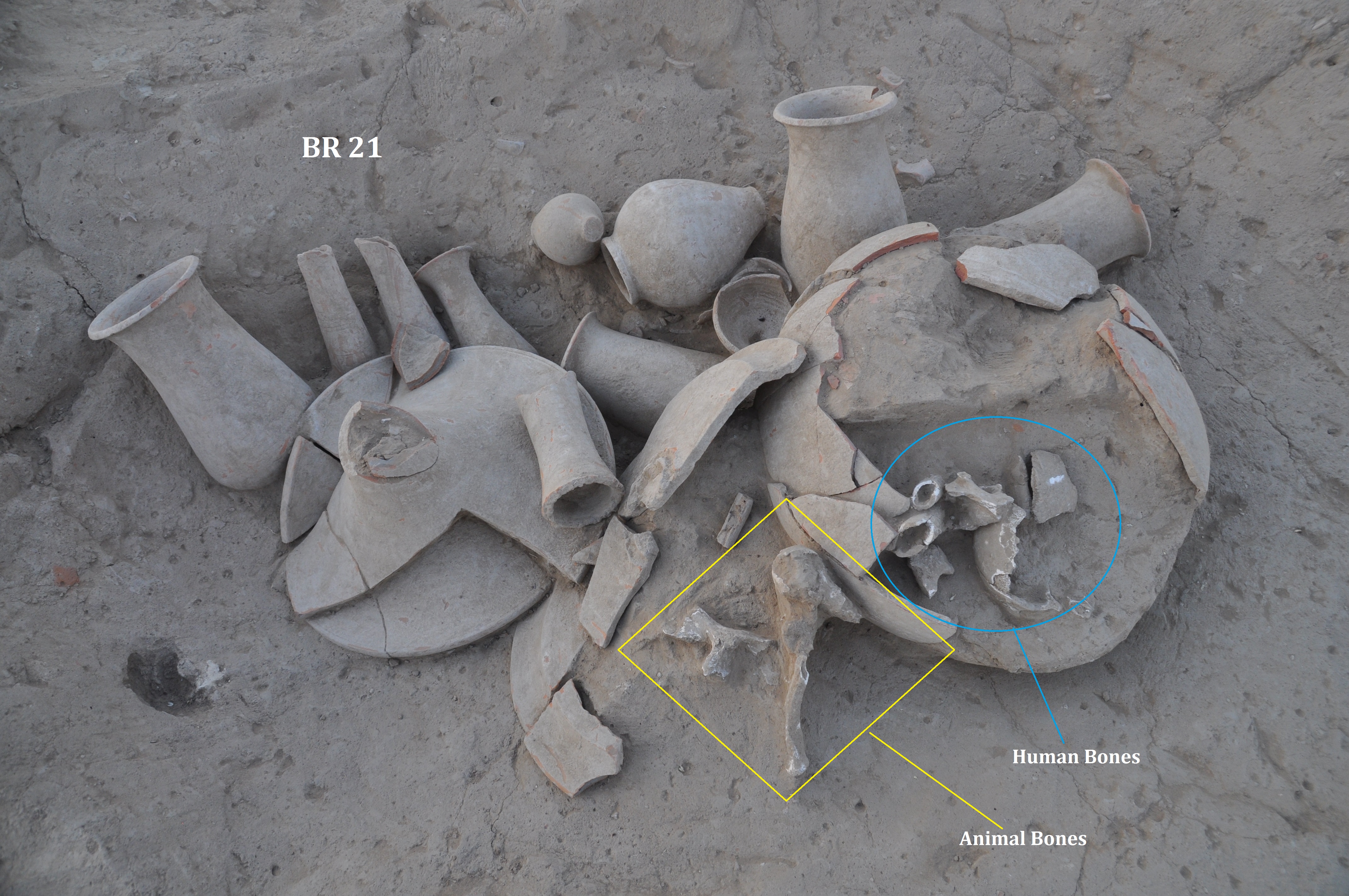

Supplement: S3 Fig — The pot burial was placed in a circular pit. Adult human skull and a few long bones were kept inside a jar. The skeletons might have been buried temporarily in one place before finally being moved for a pot burial. Note the animal bones placed on the dish. (JPG) [file pone.0192299.s003.JPG]

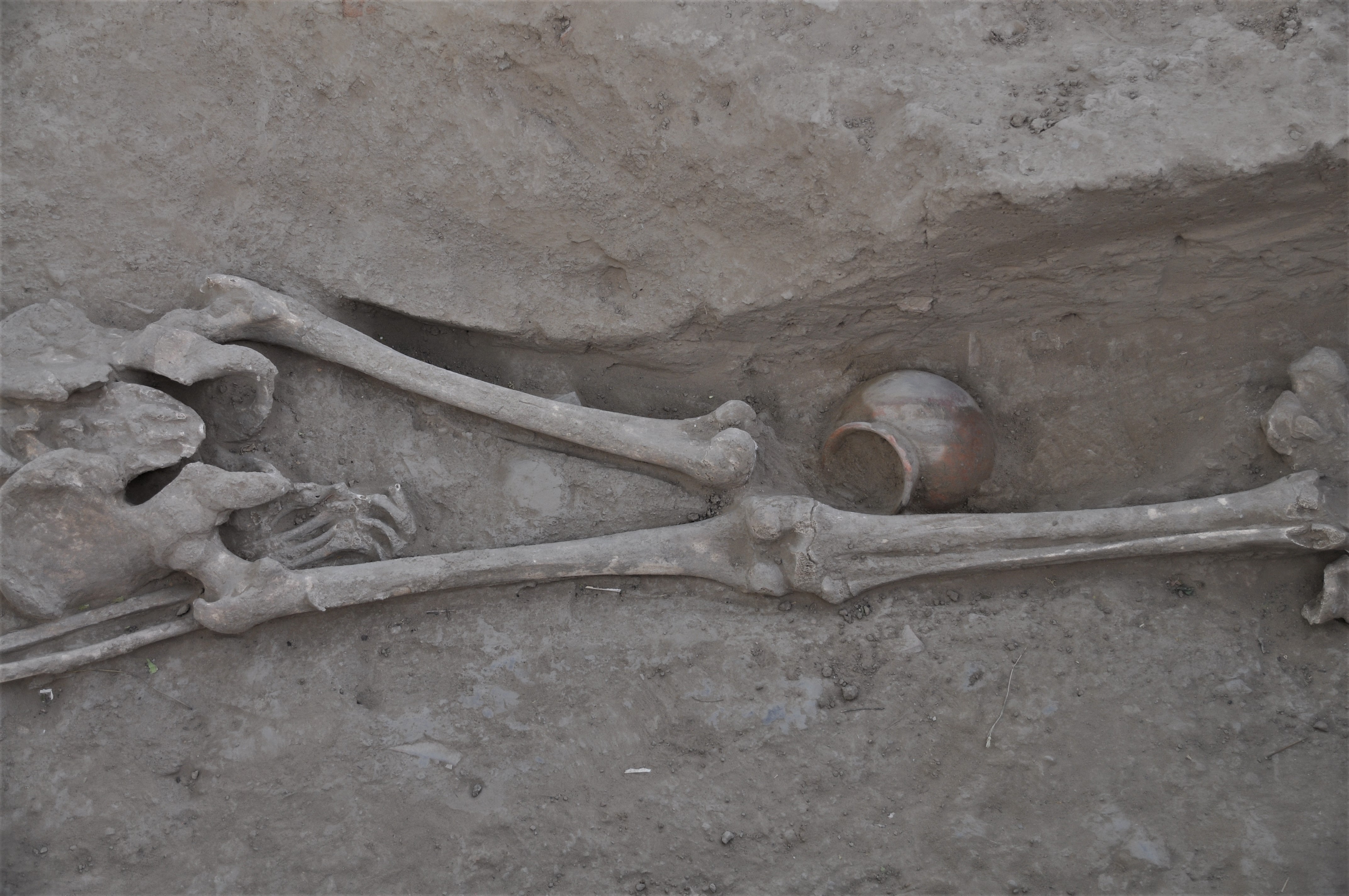

Supplement: S4 Fig — Same kind of small pots was found in the same way under individuals’ knees at two different primary burials: (A) B2/BR A1 and (B) B2/BR C1. (JPG) [file pone.0192299.s004.JPG]

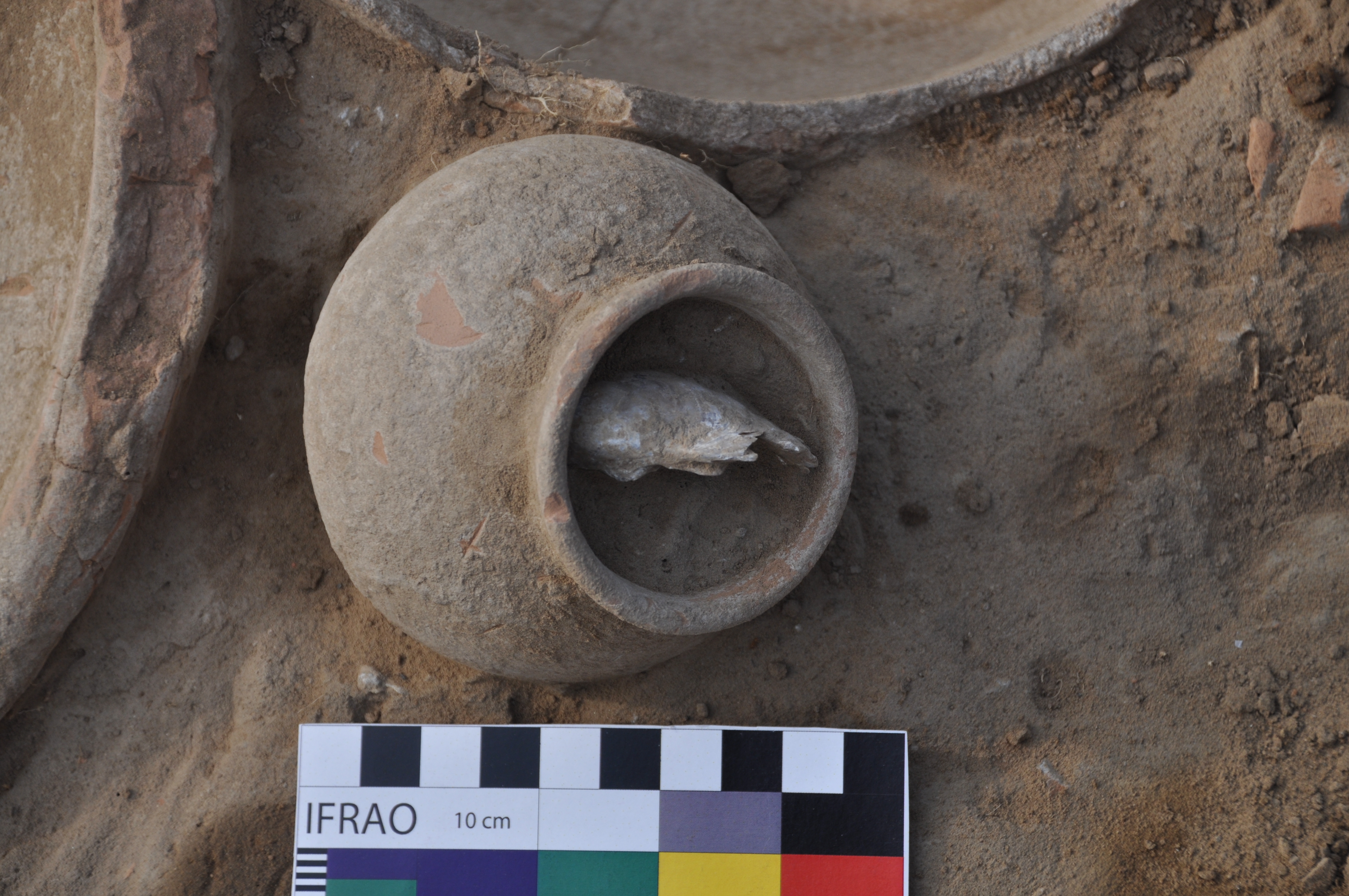

Supplement: S5 Fig — Pottery set for one individual’s grave was similar to those of two adjacent burials: (A) A2/BR13 and (B) A2/BR 15. The shell spoons were found inside the small pots. (JPG) [file pone.0192299.s005.JPG]

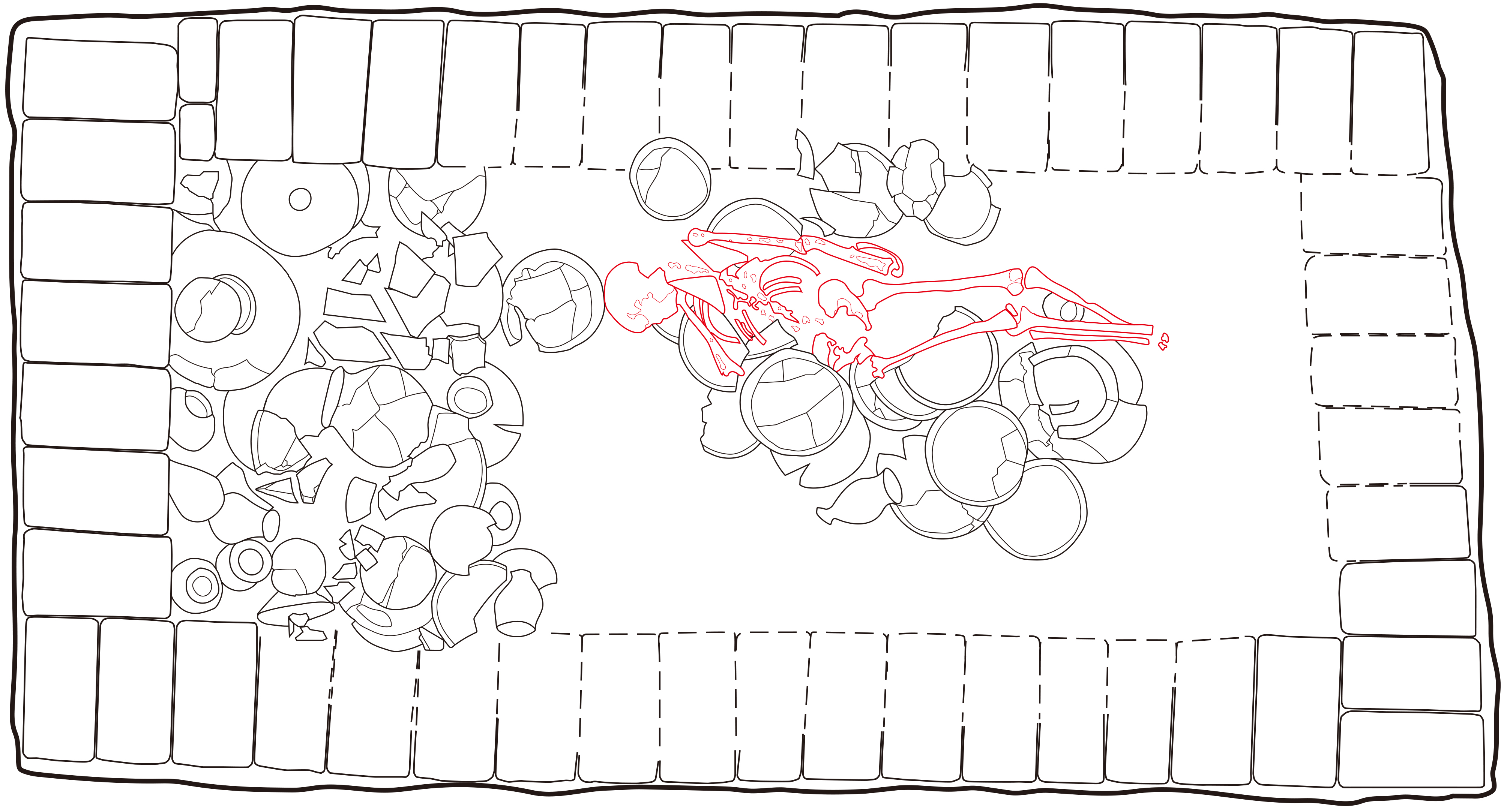

Supplement: S6 Fig — The grave structure of this burial is very similar to our Rakhigahi A2/BR33 case. The figure is here redrawn from the original of the previous report [8]. (JPG) [file pone.0192299.s006.jpg]
